# Cooperation between host immunity and the gut bacteria is essential for helminth-evoked suppression of colitis

**DOI:** 10.1186/s40168-021-01146-2

**Published:** 2021-09-13

**Authors:** Adam Shute, Blanca E. Callejas, ShuHua Li, Arthur Wang, Timothy S. Jayme, Christina Ohland, Ian A. Lewis, Brian T. Layden, André G. Buret, Derek M. McKay

**Affiliations:** 1 Gastrointestinal Research Group, Inflammation Research Network and Host-Parasite Interaction Group, Calvin, Phoebe & Joan Snyder Institute for Chronic Diseases, Department of Physiology and Pharmacology, Cumming School of Medicine, University of Calgary, Calgary, Alberta Canada; 2 International Microbiome Center, Cumming School of Medicine, University of Calgary, Calgary, Canada; 3 Department of Biological Sciences, Faculty of Science, University of Calgary, Calgary, Canada; 4 Division of Endocrinology, Diabetes, and Metabolism, University of Illinois at Chicago, Chicago, IL USA; 5 Jesse Brown Veterans Affairs Medical Center, Chicago, IL USA

## Abstract

**Background:**

Studies on the inhibition of inflammation by infection with helminth parasites have, until recently, overlooked a key determinant of health: the gut microbiota. Infection with helminths evokes changes in the composition of their host’s microbiota: one outcome of which is an altered metabolome (e.g., levels of short-chain fatty acids (SCFAs)) in the gut lumen. The functional implications of helminth-evoked changes in the enteric microbiome (composition and metabolites) are poorly understood and are explored with respect to controlling enteric inflammation.

**Methods:**

Antibiotic-treated wild-type, germ-free (GF) and free fatty-acid receptor-2 (*ffar2*) deficient mice were infected with the tapeworm *Hymenolepis diminuta*, then challenged with DNBS-colitis and disease severity and gut expression of the il-10 receptor-α and SCFA receptors/transporters assessed 3 days later. Gut bacteria composition was assessed by 16 s rRNA sequencing and SCFAs were measured. Other studies assessed the ability of feces or a bacteria-free fecal filtrate from *H. diminuta*-infected mice to inhibit colitis.

**Results:**

Protection against disease by infection with *H. diminuta* was abrogated by antibiotic treatment and was not observed in GF-mice. Bacterial community profiling revealed an increase in variants belonging to the families *Lachnospiraceae* and *Clostridium* cluster *XIVa* in mice 8 days post-infection with *H. diminuta*, and the transfer of feces from these mice suppressed DNBS-colitis in GF-mice. Mice treated with a bacteria-free filtrate of feces from *H. diminuta*-infected mice were protected from DNBS-colitis. Metabolomic analysis revealed increased acetate and butyrate (both or which can reduce colitis) in feces from *H. diminuta*-infected mice, but not from antibiotic-treated *H. diminuta*-infected mice. *H. diminuta*-induced protection against DNBS-colitis was not observed in *ffar2*^*−/−*^ mice. Immunologically, anti-il-10 antibodies inhibited the anti-colitic effect of *H. diminuta*-infection. Analyses of epithelial cell lines, colonoids, and colon segments uncovered reciprocity between butyrate and il-10 in the induction of the il-10-receptor and butyrate transporters.

**Conclusion:**

Having defined a feed-forward signaling loop between il-10 and butyrate following infection with *H. diminuta*, this study identifies the gut microbiome as a critical component of the anti-colitic effect of this helminth therapy. We suggest that any intention-to-treat with helminth therapy should be based on the characterization of the patient’s immunological and microbiological response to the helminth.

**Supplementary Information:**

The online version contains supplementary material available at 10.1186/s40168-021-01146-2.

## Introduction

Despite significant increases in therapeutics for chronic inflammatory disease, even the best of these (e.g., anti-TNF*α* antibody) is ineffective in a substantial number of patients. The rapidity of the emergence and increase in incidence of idiopathic auto-inflammatory disease supports a role for environmental factors in the pathogenesis of these conditions [1, 2]: an awareness that can direct the search for new therapeutic approaches. The inverse correlation between the geographical distribution of inflammatory bowel disease (IBD), diabetes, and multiple sclerosis with endemic parasitic helminth-infections has led to the hypothesis that infection with helminths could confer protection against auto-inflammatory disease [3]. A position supported by the fact that helminths have evolved to manipulate their hosts’ immune system [4, 5]. Indeed, analyses of animal models show that infection with helminth parasites reduces the severity of inflammatory disease [6–11], in which interleukin (il)-10, transforming growth factor (tgf)-*β*, and regulatory T cells, B cells, and macrophages were critical host factors in the inhibition of inflammation [12–16].

This immune-centric view of the host-parasite interaction overlooks the possible, if not probable, participation of the microbiome in a tripartite relationship. Descriptions of increased bacterial species richness or diversity in helminth-infected rodents and people are common [17–21], but the functional consequences of these changes in the microbiome to gut homeostasis are not well understood. The juxtaposition of helminth and bacteria in the gut allows for the possibility that the anti-inflammatory effect that follows infection with the parasite could, at least in part, be via the microbiota. This postulate is supported by data showing that reduced airways inflammation in mice infected with the nematode *Heligmosomoides polygyrus* was abrogated by antibiotic treatment [22, 23].

The mouse is a non-permissive host for the rat-tapeworm, *Hymenolepis diminuta*. Lacking hooks or teeth, this helminth does minimal damage to the host and seeks to establish in the small intestine (it does not migrate through the host): the mouse mounts a Th2-dominated immune response and expels the worm within 8–11 days of a primary infection [24]. Infection with *H. diminuta* reduces the severity of dinitrobenzene sulphonic acid (DNBS)-induced colitis in mice, and il-10 is important in this event [6]. *H. diminuta*-infection caused subtle, yet distinct, changes in the composition of the mouse colonic microbiota, but the bacteria were not required for expulsion of the worm [25]. This presents a model to address the issue of the intersection of helminths and gut bacteria in the regulation of colitis. The data herein, show that host immunological and microbiota responses (i.e., increased short-chain fatty acids (SCFAs) synthesis) are essential to the suppression of colitis initiated by infection with *H. diminuta*. Thus, in the development of new approaches to inflammatory disease, these data suggest that helminth therapy may be rendered ineffective in an individual with a reduced capacity to make il-10 (which may be a rare occurrence) or with gut dysbiosis.

## Results

### Antibiotic (Abx) treatment abrogates *H. diminuta*-evoked suppression of colitis

**Fig. 1:**
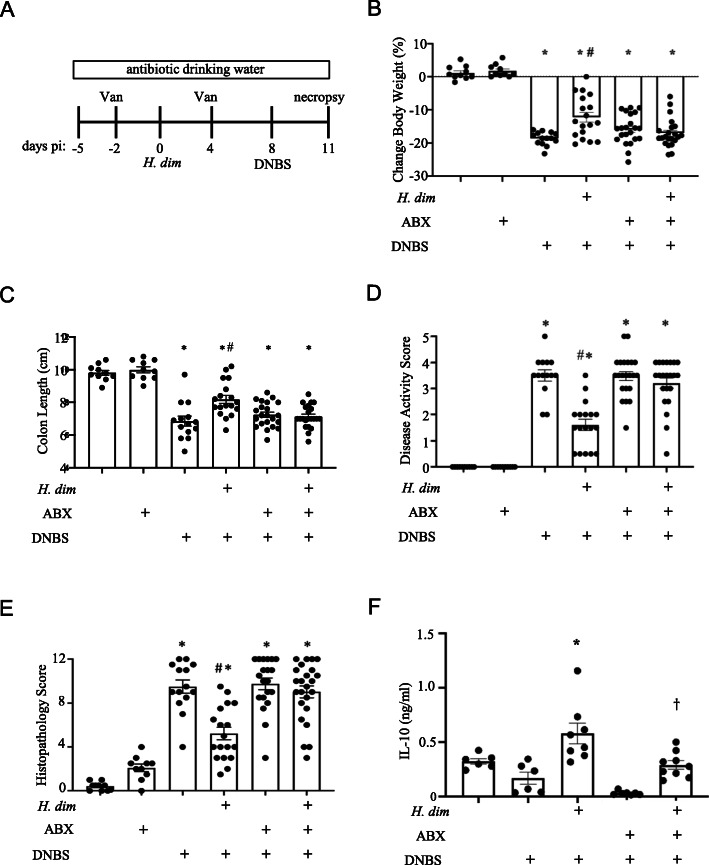
Broad-spectrum antibiotic treatment prevents *H. diminuta*-evoked inhibition of colitis. Male BALB/c mice were treated as shown in panel **A** (*H. dim*, *H. diminuta* 5 cysticercoids orally; DNBS, 3 mg ir.; ABX-drinking water *ad libitum*), and 3 days after DNBS, disease severity was assessed by **B** change in body weight, **C** colon length, **D** disease activity score, and **E** histopathology score (representative H&E images in suppl. Fig. 1). Panel **F** shows il-10 production by conA-stimulated splenocytes (2 μg/mL, 5 × 10^6^ cell/mL, 48 h) (data are mean ± SEM combined data from 3 to 4 experiments (data in panel **F** are from 2 experiments); ABX, antibiotic cocktail of kanamycin (40 mg/L), gentamicin (3.5 mg/L), colistin (4.2 mg/L), and metronidazole (21.5 mg/L); vancomycin (Van), 200 μL of 0.5 mg/mL by intraperitoneal injection; *, #,† *p* < 0.05 compared to control, DNBS and DNBS+ABX, respectively; pi, post-infection)

The possibility that the gut bacteria participated in *H. diminuta*-evoked suppression of colitis was tested with broad-spectrum antibiotics (Fig. 1A). As assessed by body weight, colon length and disease and histopathology scores, the suppression of DNBS-induced colitis evoked by infection with *H. diminuta* was absent in mice co-treated with antibiotics (ABX) (Fig. 1B–E and Suppl. Fig. 1). ConA-stimulated splenocytes (used as a marker of systemic immunity and a surrogate to confirm successful infection) from *H. diminuta*+DNBS-treated mice produced more il-10 than those from non-infected or DNBS-only treated mice (Fig. 1F). The magnitude of the splenic il-10 production from ABX+*H. diminuta*+DNBS-treated mice was reduced, yet was significantly greater than that produced by splenocytes for ABX+DNBS-treated mice (Fig. 1F).

**Fig. 2:**
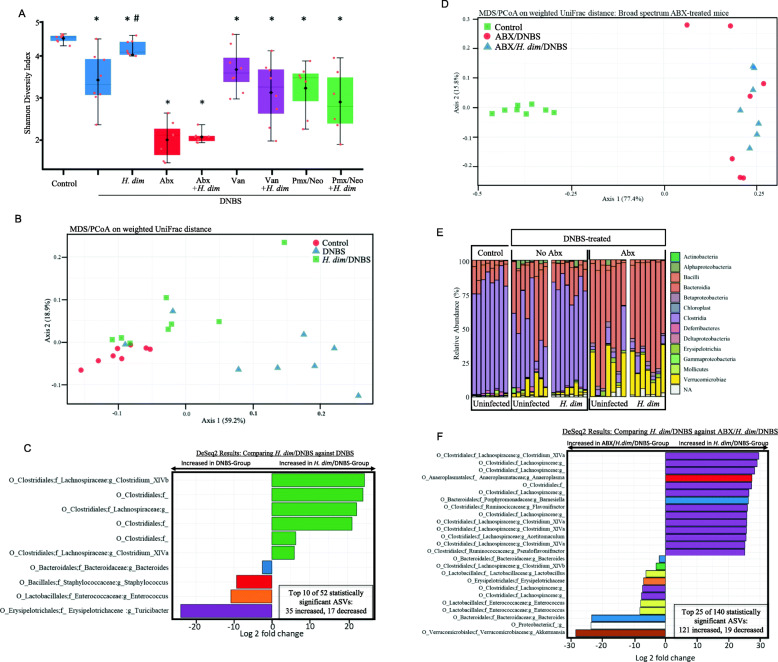
*H. diminuta* preservation of the gut microbiota in DNBS-treated mice is overcome by broad-spectrum antibiotic treatment (ABX). Male BALB/c mice were treated as shown in Fig. 1A (5 cysticercoids of *H. diminuta* (*H. dim*) 8 days prior to di-nitrobenzene sulphonic acid (DNBS; 3 mg, ir) with necropsy 3 days later ± ABX) and colon-associated bacteria assessed by 16s rRNA sequence analysis. **A** Reduced Shannon index (*α*-diversity) caused by DNBS-colitis was significantly prevented by *H. diminuta*-infection, while ABX-treatment, independent of DNBS or *H. diminuta* had the greatest impact on *α*-diversity. **B** *β*-diversity (PCoA; Weighted UniFrac distance) reveals separation of the groups with control and *H. dim*+DNBS clustering away from DNBS-only treated mice and characterized by increased ASVs for *Lachnospiraceae*, Clostridales, and *Clostridium-XIVa* (**C**). **D–F** UniFrac distance and relative abundance analyses show the impact of ABX on colonic microbiota of DNBS ± *H. diminuta*-treated mice. (ABX, antibiotic cocktail in drinking water *ad libitum*, kanamycin (40 mg/L), gentamicin (3.5 mg/L), colistin (4.2 mg/L), metronidazole (21.5 mg/L), and ip. vancomycin (van) at 200 μL of 0.5 mg/mL; panel **A**: Pmx/Neo, polymyxin B (1 g/L) and neomycin (500 mg/L) in drinking water; horizontal line, median; black diamond, mean; box plots, 25–75% quartiles; vertical line, minimum and maximum value; Mann-Whitney *U* test; see suppl. Fig. 3A and D for treatment protocol)

Profiling of the bacterial composition revealed a lower Shannon index in colon-associated bacteria from DNBS-treated mice compared to control, with *H. diminuta*+DNBS-treated mice having an intermediate phenotype, statistically different from the other two groups (Fig. 2A). A similar pattern was noted for *β*-diversity, with the exception that two mice in the DNBS group clustered with controls; these mice had the lowest disease scores in the DNBS group (Fig.2B). Differential abundance analysis revealed significant increases in ASVs in the family *Lachnospiraceae* (p=2.92 × 10^-14^) and the *Clostridium* clusters *XIVa* (*p* = 1.24 × 10^−4^) and *XIVb* (*p*=1.94 × 10^−16^) in *H. diminuta*+DNBS treated mice compared to DNBS-only treatment (Fig. 2C). DNBS-only treated mice had increased variants belonging to the families *Bacteroidaceae* (*p*=3.05 × 10^−5^), *Staphylococcaceae* (*p*=1.60 × 10^−7^), *Enterococcaceae* (*p* = 3.39 × 10^−5^), and *Erysipelotrichaceae* (*p* = 1.15 × 10^−16^) compared to the *H. diminuta*+DNBS group (Fig. 2C). As expected, mice treated with ABX (broad spectrum, vancomycin only, or polymyxin B + neomycin ± DNBS ± *H. diminuta*) displayed severe disruption of their microbiota (Fig. 2A, D–F), with a general shift away from Firmicutes and to Bacteroidetes (Fig. 2F). Differential abundance analysis identified significant increases in ASVs belonging to the genus *Akkermansia* (*p* = 1.11 × 10^−21^), *Enterococcus* (*p* = 6.46 × 10^−5^), and *Bacteroides* (*p* = 3.96 × 10^−16^), as well as the phylum Proteobacteria (*p* = 2.16 × 10^−15^) in *H. diminuta*+DNBS+ABX compared to *H. diminuta*+DNBS-treated mice (Fig. 2F). Sequence variants belonging to *Lachnospiraceae* were significantly (*p* = 4.02 × 10^−23^) depleted in ABX+DNBS-treated mice.

Use of different antibiotics (vancomycin to target Gram-positive bacteria, polymyxin B+neomycin to target Gram-negative bacteria) to modulate microbiota composition significantly reduced the richness of the murine gut microbiota (Fig. 2A; Suppl. Fig. 2). Treating *H. diminuta*-+DNBS mice with vancomycin resulted in the reduction of several ASVs, specifically those belonging to the family *Lachnospiraceae* (*p* = 2.53 × 10^−14^) and the *Clostridia* cluster *XIVb* (*p* = 9.47 × 10^−18^) compared to the *H. diminuta*+DNBS group (some *Lachnospiraceae* ASV were increased in the *H. diminuta*+DNBS+vancomycin group and so additional sequencing will be needed to identify the species that differ in the two groups). Similarly, polymyxin B+neomycin-*H. diminuta*+DNBS-treated mice lacked variants belonging to the *Lachnospiraceae* family (*p* = 7.34 × 10^−20^) and the *Clostridium* cluster *XIVa* (*p* = 2.67 × 10^−21^) compared to the *H. diminuta*+DNBS group (Suppl. Fig. 2). The impact of either vancomycin or polymyxin B+neomycin on the ability of *H. diminuta* to suppress DNBS-induced colitis was variable, such that disease and histopathology scores were not statistically different from the DNBS or *H. diminuta*+DNBS groups (Suppl. Fig. 3), and likely reflects the composition of the microbiota in individual mice at the start of the experiment combined with the variable response to DNBS. Splenocytes from *H. diminuta*+DNBS+vancomycin or *H. diminuta*+DNBS+polymyxin B+neomycin-treated mice produced levels of il-10 that were not different from control, and in contrast to significantly increased output of il-10 from conA-stimulated splenocytes for *H. diminuta*+DNBS-treated mice (Suppl. Fig. 4).

Increased splenocyte il-10 production from GF-mice confirmed a response to infection with *H. diminuta* (Suppl. Fig. 5A) [25]. GF-mice infected with *H. diminuta* had increased colonic il-10 mRNA compared to control, while il-10r*α* mRNA levels were not different from uninfected GF-mice (Suppl. Fig. 5B). While the severity of DNBS-induced colitis was variable in GF-mice, infection with *H. diminuta* did not elicit a significant anti-colitic effect in these mice (Suppl. Fig. 5C, D).

### Fecal microbial transplants from *H. diminuta*-infected mice inhibits colitis

**Fig. 3:**
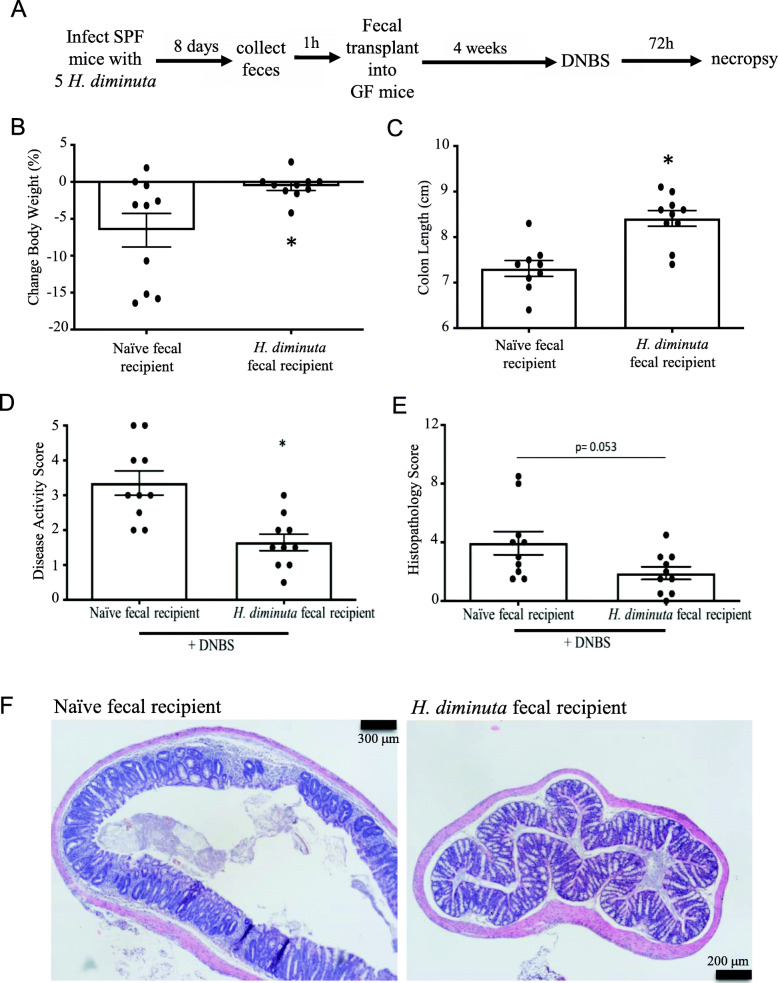
Feces from *H. diminuta*-infected mice protects mice from DNBS-treated mice. **A** Experimental paradigm of treatment of male BALB/c germ-free (GF)-mice with feces from mice infected with 5 *H. diminuta* 8 days previously. Di-nitrobenzene sulphonic acid (DNBS: 3 mg, ir.)-induced colitis evoked 4 weeks after fecal microbial transplant was assessed by body weight (**B**), colon length (**C**), and disease (**D**) and histopathology scores (**E**). Panel **F** show representative H&E stained sections of mid-colon (data are mean ± SEM combined from 2 experiments; *, *p* < 0.05 compared to mice receiving feces from naive control mice; SPF, specific pathogen-free)

Fresh feces were collected from control specific pathogen-free (SPF)-mice and mice infected with *H. diminuta* 8 days previously, processed under anaerobic conditions, and gavaged into separate groups of GF-mice (Fig. 3A); animals that received feces from *H. diminuta*-infected donor mice had less severe colitis when challenged with DNBS 4 weeks later (Fig. 3B–F).

Analysis revealed bacterial community compositions in feces from control and *H. diminuta*-infected mice consistent with our previous observations (data not shown) [25], with a small increase in *α*-diversity in the infected mice (Suppl. Fig. 6A). Four weeks post-colonization, *α*-diversity was not different between the groups, whereas taxonomic *β*-diversity as determined by weighted Unifrac distance showed distinct separation of the groups (Suppl. Fig. 6B), that was still apparent on necropsy 72 h after DNBS-treatment (Suppl. Fig. 6A,B). Differential abundance analysis revealed greater abundance of ASVs belonging to the family *Lachnospiraceae* (*p* = 1.33 × 10^−27^) and *Clostridia* cluster *XIVa* (*p* = 4.94 × 10^−14^) in feces from *H. diminuta*-infected donors compared to that from naïve-donor SPF-mice (Suppl. Fig. 6C). At 4 weeks post-colonization, mice that received feces from *H. diminuta*-infected mice had a higher abundance of *Lachnospiraceae* (*p* = 8.97 × 10^−13^), *Ruminococcus* (*p* = 8.46 × 10^−25^), and *Clostridium* cluster *XIVa* (*p* = 6.32 × 10^−13^), while ASVs assigned to the families *Clostridiaceae_1* (*p* = 3.67 × 10^−15^) and *Ruminococcaceae* (*Flavonifractor*; *p* = 2.83 × 10^−14^), and *Clostridium* cluster *IV* (*p* = 5.62 × 10^−14^) were increased in mice that received control donor feces (Suppl. Fig. 6D). Finally, differential abundance analysis showed significant increases in ASVs belonging to the families *Lachnospiraceae* (*p* = 2.12 × 10^−14^) and *Ruminococcaceae* (*p* = 3.11 × 10^−17^), as well as *Clostridium* cluster *XIVa* (*p* = 1.22 × 10^−34^) in DNBS-treated mice that received feces from *H. diminuta*-donors compared to DNBS-treated mice that received feces from naïve-donors; the latter demonstrated substantial increases within the families *Enterobacteriaceae* (*p* = 4.75 × 10^−37^) and *Bacteroidaceae* (*p* = 2.89 × 10^−41^) (Suppl. Fig. 6E).

### Feces from *H. diminuta*-infected mice have increased SCFA

**Fig. 4:**
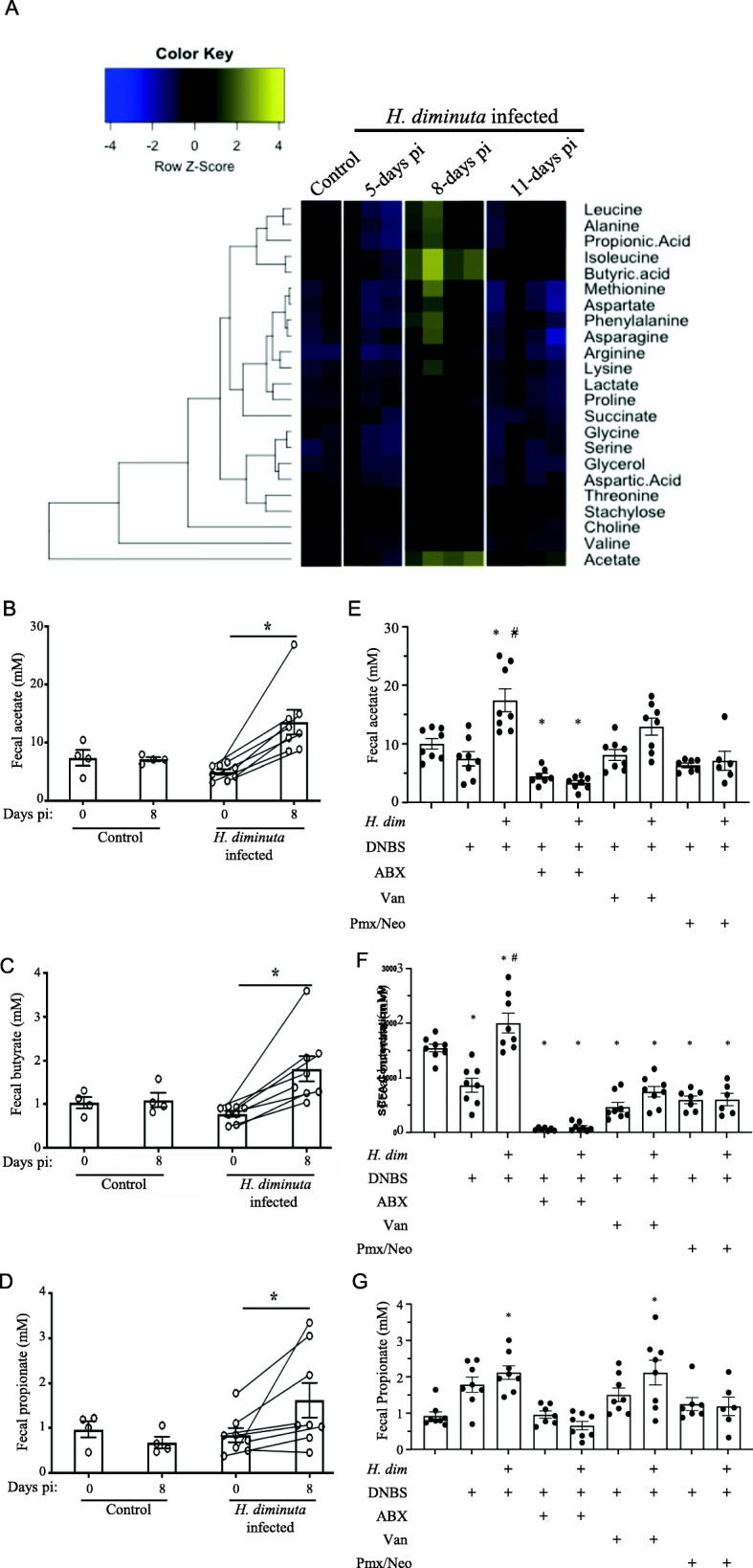
Feces from *H. diminuta*-infected mice contain increase amounts of short-chain fatty acids (SCFA). **A** Heat-map of NMR results shows increased acetate, propionic acid and butyric acid in feces from mice infected 8 days previously with *H. diminuta*. **B–D** Separate analyses confirmed increased SCFA in feces of infected mice (paired *t* test, day 0 vs. 8 days post-infection). **E–G** Broad-spectrum antibiotic treatment (ABX) (see Fig. 1A) prevented the *H. diminuta* (*H. dim*) evoked increase in fecal acetate or butyrate, and a similar pattern was observed in mice treated with vancomycin (Van) or polymyxin B and neomycin (Pmx/Neo) (see Suppl. Fig. 3A,D) (data are mean ± SEM; *, *p* < 0.05 compared to control or between indicated groups; #, *p* < 0.05 compared to DNBS (di-nitrobenzene sulphonic acid, 3 mg, ir., necropsy 72 post-DNBS; *H. dim*, 5 cysticercoids 8 days prior to DNBS; pi, post-infection)

Initial NMR analyses revealed increased acetate, propionate, and butyrate in feces from 8-day *H. diminuta*-infected mice compared to non-infected mice (Fig. 4A). The increases were transitory and were not seen with this technique when feces from 11-dpi with *H. diminuta* were assessed (Fig. 4A). These increases in SCFAs were confirmed by paired LC-MS analyses on feces from individual mice collected prior to and 8 days post-infection with *H. diminuta* (Fig. 4B–D). The increased levels of acetate, butyrate, and propionate in feces from *H. diminuta*-infected mice were ablated by antibiotic co-treatment, particularly the cocktail with broad-spectrum activity (Fig. 4E–G). The anti-colitic effects of butyrate and acetate were confirmed by enema delivery or continuously in the drinking water, respectively (Suppl. Fig. 7).

### Bacteria-free filtrate of feces from *H. diminuta*-infected mice reduces DNBS-induced colitis

**Fig. 5:**
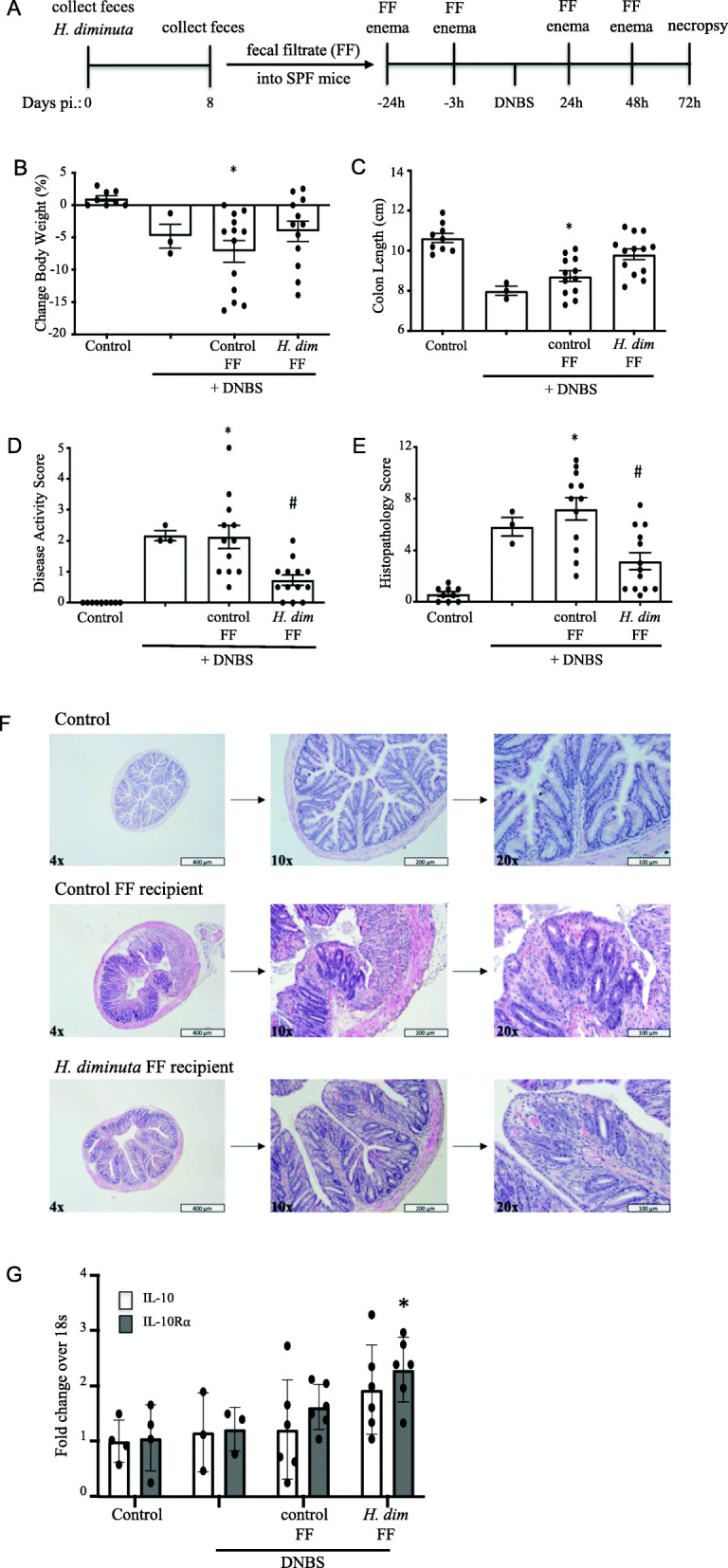
Bacteria-free filtrate of feces from *H. diminuta*-infected mice reduces the severity of DNBS-induced colitis. Feces was collected from *H. diminuta*-infected mice and passed through a 0.2 μm filter (FF) and administered to specific-pathogen-free male BALB/c mice as shown in panel **A**. Seventy-two hours after di-nitrobenzene sulphonic acid (DNBS: 3 mg, ir.), disease severity was assessed by **B** body weight, **C** colon length, **D** disease activity, and **E** histopathology scores. Representative H&E stained sections of mid-colon are shown in panel **F**. Panel **G** shows q-PCR for il-10 and il-10r*α* in colonic segments from FF-treated mice (data are mean ± SEM combined data from 2 experiments; * and #, *p* < 0.05 compared to control and control fecal filtrate from naive non-infected mice, respectively; *H. dim* FF, fecal filtrate from *H. diminuta*-infected (5 cysticercoids) mice; pi, post-infection)

Intra-rectal delivery of a fecal filtrate (FF) from day-8 *H. diminuta*-infected mice four times over the course of DNBS-induced colitis (Fig. 5A) significantly reduced the severity of disease (Fig. 5B–F), as gaged by disease and histopathology scores, but not body weight. Mice that received control FF or FF from *H. diminuta*-infected donors had longer controls than DNBS-only treated mice, suggesting a mild benefit of fecal filtrate in this model system: the benefit was most pronounced with the FF from infected mice. Colonic tissue from mice that received the FF from *H. diminuta*-infected mice had increased il-10r*α* mRNA compared to those receiving FF from naïve donor mice, but il-10 mRNA was not statistically significantly increased (Fig. 5G). The FF from *H. diminuta*-infected mice contained more acetate (4.2±0.81 mM*) and butyrate (467±90 μM*) compared to FF from naïve mice (acetate = 2.3±0.53 mM; butyrate = 272±52 μM; *n* = 3; *, *p* < 0.05 unpaired *t* test).

### DNBS-induced colitis in ffar2^−/−^ mice is not affected by infection with *H. diminuta*

**Fig. 6:**
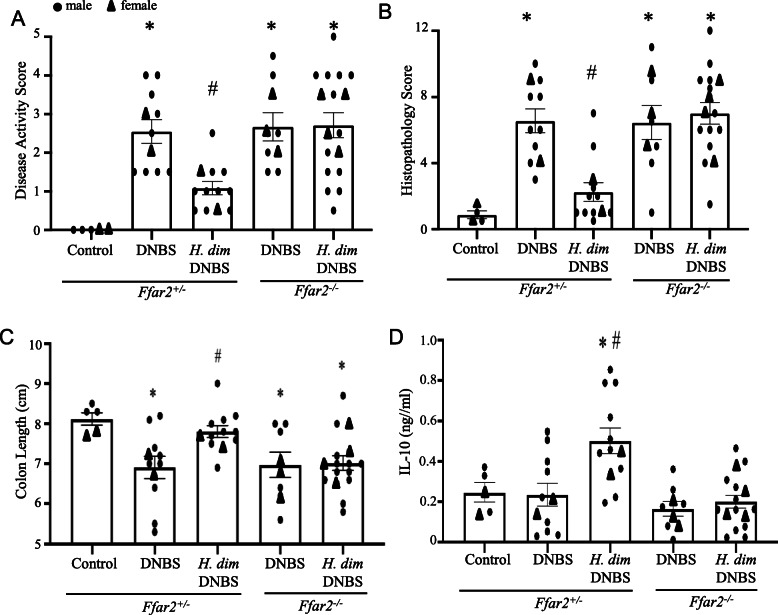
Free-fatty acid repector-2 knock-out mice are not protected from DNBS-induced colitis by infection with *H. diminuta*. Male *ffar2*^*−/−*^ mice and *ffar2*^*+/−*^ littermates were infected with 5 cysticercoids of *H. diminuta* (*H. dim*) and 8 days later were challenged with di-nitrobenzene sulphonic acid (DNBS, 3 mg, ir). At necropsy 72 h post-DNBS, disease was assessed by **A** disease activity and **B** histopathology scores and colon length (**C**). **D** Isolated splenocytes (5 × 10^6^/mL) were stimulated with concanavalin-A (2 μg/ml) for 48 h and il-10 production measured by ELISA (data are mean ± SEM combined data from 2 experiments; * and #, *p* < 0.05 compared to control and DNBS-only in the matched mouse strain)

C57/Bl6 free-fatty acid (*ffar)-2*^*+/*−^ mice infected with *H. diminuta* were protected from DNBS-induced colitis, whereas infected *ffar2*^*−/−*^ littermates displayed colitis that was not significantly different from DNBS-only treated *ffar2*^*−/−*^ mice as assessed by disease activity scores, histopathology scores, and colon length (Fig. 6A–C). The anti-colitic effect of infection with *H. diminuta* observed in *ffar-2*^*+/−*^ was accompanied by increased il-10 production by conA-stimulated splenocytes compared to splenocytes from naïve *ffar-2*^*+/−*^ mice and *H. diminuta*+DNBS treated *ffar2*^*−/−*^ mice (Fig. 6D).

### Anti-il10 antibodies eliminate the anti-colitic effect of infection with *H. diminuta*

Consistent with previous findings [6], the inhibition of DNBS-induced colitis by infection with *H. diminuta* was abrogated in mice treated with neutralizing anti-il-10 antibodies, as assessed by disease activity and histopathology scores (Suppl. Fig. 8).

### Reciprocal regulation of il-10 receptor and butyrate transporters/receptors

**Fig. 7:**
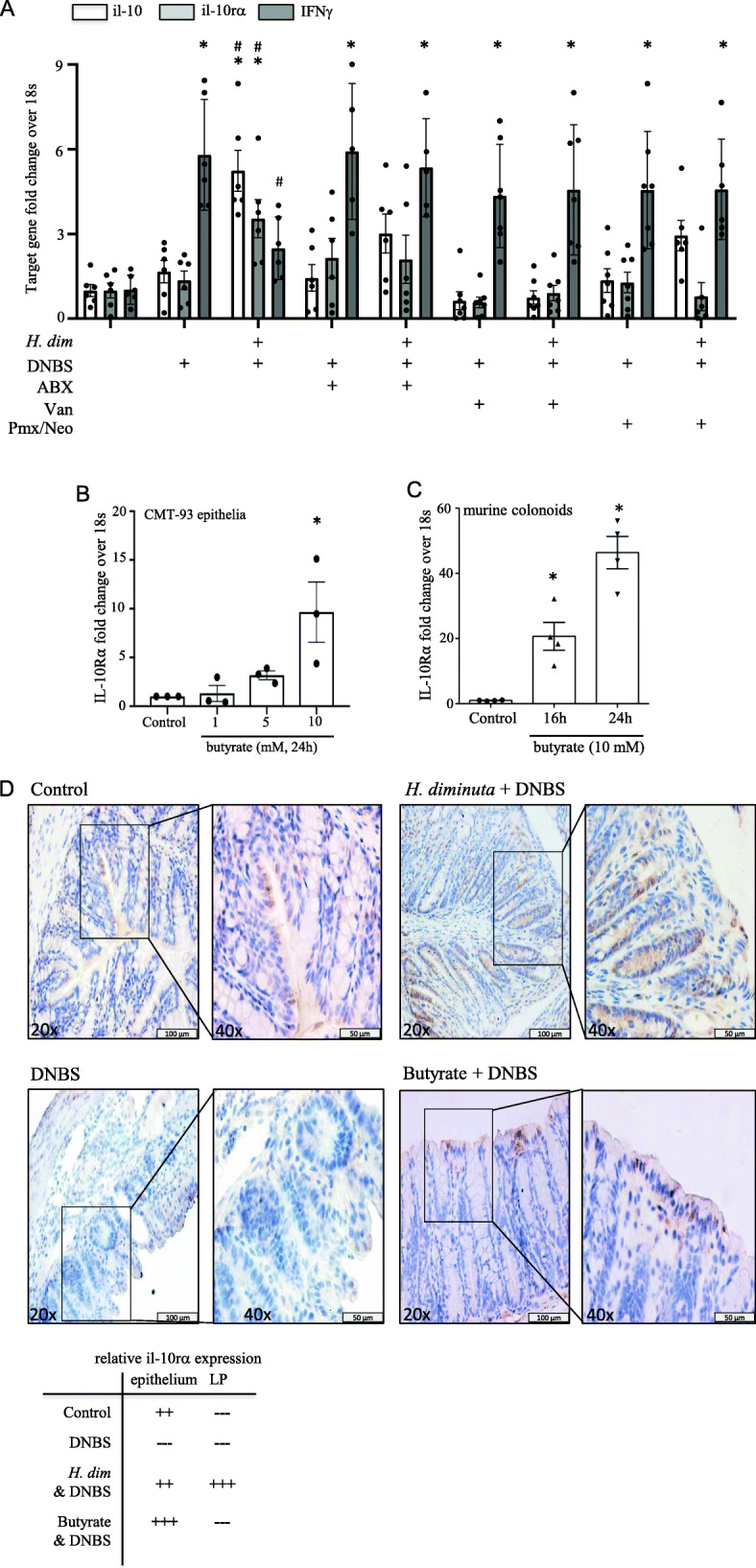
Helminth infection and butyrate upregulates IL-10 receptor expression. qPCR reveals increased in il-10 and il-10 receptor-*α* and reduced interferon (ifn)-*γ* in mid-colon of *H. diminuta* and DNBS treated mice compared to control and DNBS only treated mice. Co-treatment with broad-spectrum antibiotics (ABX), vancomycin (Van), or a mixture of polymyxin B (Pmx) and neomycin (Neo) prevented the increase in il-10 or il-10 receptor-α (rα) mRNA. Exposure to butyrate (1-10 mM; 16–24 h) significantly increased the expression of il-10r*α* mRNA in **B** a murine rectal epithelial cell line and **C** primary murine colonoids. Panel **D** is a qualitative assessment of il-10r*α* expression on sections of mid-colon as detected by immunocytochemistry, with representative images depicted (data are mean ± SEM; *, *p* < 0.05 compared to control; *H. dim*, mice infected with 5 *H. diminuta* 8 days before intra-rectal di-nitrobenzene sulphonic acid (DNBS, 3 mg, 72 h); butyrate (500 micro-L of 100 mM enema was delivered 24 and 3 h before DNBS and 24 and 48 h after DNBS) (ABX, antibiotic cocktail in drinking water *ad libitum*, kanamycin (40 mg/L), gentamicin (3.5 mg/L), colistin (4.2 mg/L), metronidazole (21.5 mg/L); ip. Van. at 200 μL of 0.5 mg/mL; Pmx/Neo, 1 g/L and 500 mg/L in drinking water (see Fig. 1A and suppl. Fig. 3A,D for treatment protocols)

Colonic tissue excised from *H. diminuta*+DNBS treated mice displayed increased il-10 and il-10r*α* mRNA and decreased IFN*γ* mRNA compared to DNBS-only treated mice: these changes were abrogated by antibiotic treatment of *H. diminuta*+DNBS treated mice (Fig. 7A). Corroborating and extending data with human gut-derived cell lines [26], we find that butyrate increases il-10r*α* mRNA expression in a mouse rectal epithelial cell line and in primary mouse colonoids in a dose- and time-dependent manner (Fig. 7B, C). Immunostaining revealed il-10r*α*-immunoreactivity in the colon of control mice that was most prominent on the apical epithelium with minimal positivity on lamina propria cells (as demonstrable by this technique), whereas tissue from DNBS-treated mice was largely devoid of il-10r*α*-immunoreactivity (Fig.7D). Colon from *H. diminuta*+DNBS treated mice had widespread il-10r*α*-immunoreactivity in the epithelium, extending deep into the crypts, and in the lamina propria (Fig.7D). Sections of colon from mice that received butyrate enemas displayed il-10r*α*-immunoreactivity that was subtly increased over that observed in control mice and was predominantly evident on the apical epithelial cells (Fig. 7D).

**Fig. 8:**
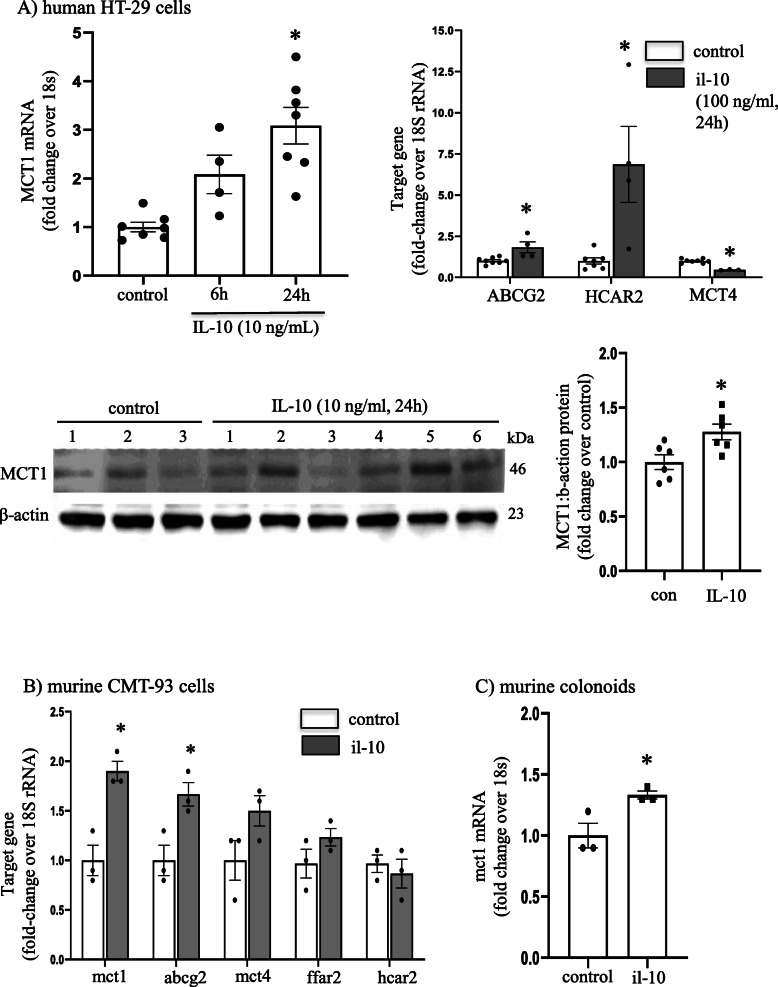
IL-10 increases short-chain fatty acid (SCFA) transporter expression. The human colon-derived HT-29 epithelial cell line treated with IL-10 (10 or 100 ng/mL, 24 h) displayed significant increases in the SCFA transporters MCT1, ABCG2, and HCAR2 mRNA and a reduction in MCT4 mRNA (**A**). The increased MCT1 mRNA was matched by a subtle increase in MCT1 protein (representative blot shown), as shown by densitometry and statistical comparisons. Panel **B** shows increased expression of mct1 and Abcg2 mRNA in the murine rectal epithelial CMT-93 cell line treated with il-10 (10 ng/ml, 24 h). IL-10 treatment evoked increased mct1 mRNA in primary murine colonoids (**C**) (10 ng/ml, 24 h) (data are mean ± SEM; data from 1 to 2 experiments; *, *p* < 0.05 compared to control; kDa, kilodaltons)

Analysis of mRNA for SCFA transporters and receptors revealed consistent induction of MCT1 mRNA in the HT-29 (Fig. 8A) and CMT-93 (Fig. 8B) epithelial cell lines and primary mouse organoids (Fig. 8C) by il-10 (HT-29 had a subtle increase in MCT1 protein) (Fig. 8A). ABCG2 mRNA was increased in il-10-treated HT-29 and CMT-93 epithelia. Il-10 treatment increased mRNA expression for the SCFA receptor HCAR (GPR109A) in HT-29 cells (Fig. 8A), while the increase in *ffar2* in CMT-93 cells failed to reach statistical significance (Fig. 8B).

## Discussion

Enthusiasm for helminth-therapy for inflammatory disease based on numerous animal model studies [27] and small clinical trials [28–31] is tempered by a lack of efficacy of *Trichuris suis* ova in larger trials [32–34]. We hypothesized that the anti-colitic effect of infection with a helminth parasite could be influenced by the gut microbiota and so its effectiveness would be reduced in IBD patients with dysbiosis [35]. The novel data herein reveal helminth, host, and gut bacteria interaction in the suppression of disease, and in untangling this tripartite mechanism of the control of enteric inflammation we note reciprocity in il-10 and butyrate signaling in the regulation of short-chain fatty acid transporter and il-10 receptor expression, respectively.

Mechanistic studies to understand how infection with helminth parasites inhibits inflammatory disease have implicated suppression of Th1 immunity or production of immunoregulatory cells and mediators [4, 13, 14, 36]. This focus on host immunological processes, while intuitive, has, until recently, overlooked the potential involvement of the host microbiota as a regulator of mucosal immunity and gut homeostasis [22, 37–39]. Following identification that infection with *H. diminuta* significantly increased bacterial species richness in mice (e.g., increased relative abundance of *Lachnospiraceae* [25] and reduced *Bacteroidaceae*, members of which may exert a pro-colitigenic effect [40]), treatment with broad-spectrum antibiotics was found to prevent the inhibition of colitis evoked by *H. diminuta*-infection. Moreover, splenocytes from the antibiotic+*H. diminuta+*DNBS-treated mice produced substantial amounts of il-10, suggesting that lack of inhibition of colitis in the antibiotic-treated mice was linked to the microbiota and not a bystander effect on the host immune response to *H. diminuta*-infection. This supposition is supported by similar severities of DNBS-induced colitis in GF-mice ± *H. diminuta*-infection. In accordance with these data, *H. polygyrus*-evoked suppression of airways inflammation or obesity induced by a high-fat diet was abrogated in mice co-treated with antibiotics [22, 41].

Dissecting the role of the microbiota in the anti-colitic evoked by helminth-infection, transfer of feces from mice infected 8 days previously with *H. diminuta* into GF-mice conferred partial, but significant, protection from DNBS-colitis. While the transfer of feces is a promising approach for some conditions [42], there are safety concerns, and microbiota from *Schistosoma mansoni-* and *H. polygyrus*-infected mice exaggerated dextran sodium sulfate- and *Citrobacter rodentium*-induced colitis, respectively [38]. The latter studies illustrate the specificity of host-parasite interaction, that infection with worms that do not inhabit the gut (i.e., *S. mansoni*) can affect the composition of the gut microbiota, and that helminth therapy for a inflammatory disease is unlikely to be via a single species of helminth [we note that differential effects of feces from helminth-infected mice may also be due to differences in the gut bacteria due to source of the animal, housing or food, and be influenced by the helminth-specific mucosal immune response]. Subsequently, enemas of filtered feces from *H. diminuta*-infected mice, but not that from uninfected mice, were found to inhibit DNBS-colitis in SPF-mice, prompting analysis of the feces for molecules that could suppress DNBS-induced colitis.

Feces from *H. diminuta-*infected mice had increased levels of the short-chain fatty acids (SCFAs), acetate and butyrate compared to uninfected mice, compatible with the increased abundance of actinobacteria and *Clostridium* cluster *XIVa.* Some, not all, individuals with IBD have benefited from butyrate enemas [43] and acetate and butyrate can be anti-inflammatory in murine models of colitis [44–46]; findings we recapitulated with the DNBS-model of colitis. While many bacteria-derived products affect the host, the finding that the *ffar2*^*−/−*^ (or G-protein coupled receptor (GPR)-43 found on colonic epithelium and immune cells [47]) mice were not protected from DNBS-induced colitis by infection with *H. diminuta* supports further a role for SCFA in the anti-colitic effect. Similarly, fecal transplants from infected mice recapitulated the reduced hypersensitivity to house dust mite in *H. polygyrus*-infected mice; in this instance acetate and ffar3 (GPR41) mediated the protective effect [22].

The data support a mechanism whereby infection with *H. diminuta* causes increased abundance of SCFA-producing bacteria, and that increased butyrate and acetate, via ffar2*,* mediates the suppression of colitis. How then to reconcile this with immunoneutralization of il-10 blocking the anti-colitic effect of infection with *H. diminuta* (*6*) (Suppl. Fig. 8)? Positing interaction via il-10 and butyrate, *H. diminuta*-infection evoked increased il-10r*α* immunoreactivity in the colon was absent in antibiotic co-treated mice, and infected GF-mice displayed increased colonic il-10, but not il-10r*α*, mRNA. Moreover, enemas with fecal-filtrate from *H. diminuta*-infected mice or butyrate into SPF-mice both resulted in an increase in colonic il-10-r*α* mRNA or protein. Butyrate directly increased il-10r*α* mRNA expression in a murine rectal epithelial cell line and primary epithelia, extending similar observations in human colon-derived epithelial cells lines [26]. Reciprocally, il-10 increased mRNA expression for one or more butyrate transporter/receptor in the human colonic HT-29 epithelial cell line, and murine CMT-93 epithelial cells and colonoids.

Butyrate and il-10 exert a range of anti-inflammatory effects [48, 49]. Thus, we speculate that infection with *H. diminuta* creates a positive feedback loop whereby bacteria-derived butyrate and host-derived il-10 cooperate to drive the anti-colitic effect: absence of either negates the beneficial effect of infection with the helminth. In accordance, the recruitment of il-10^+^ regulatory T cells to the lungs of *H. polygyrus*-infected mice was dependent on ffar3, and *H. polygyrus*-evoked changes in the gut microbiome that reduced obesity in high-fat diet-fed mice were dependent of signal transducer and activator of transcription (STAT)-6 (i.e., il-4/il-13 signaling) [50]. These findings combined with the current data illustrate the intertwined nature of the helminth-host-bacteria relationship and the interplay between host immune factors and bacteria-derived molecules in the suppression of disease.

## Conclusion

The present study advances understanding of helminth-regulation of inflammatory disease, providing evidence for a critical role of bacteria-derived SCFAs operating via ffar2 in *H. diminuta*-amelioration of colitis, the essential requirement of il-10 that can up-regulate expression of SCFA transporters/receptors, and butyrate regulation of il-10 receptor expression. Moreover, it provides one possible explanation for the lack of efficacy of helminth-therapy in recent IBD trials, such that patients who lack SCFA-producing bacteria [51], lack butyrate transporters or receptors [52], or with a diminished capacity to express il-10 or the il-10-receptor [53] would be contraindicated for this novel treatment. Extrapolating from this model system, we suggest that for helminth-therapy to be beneficial it needs to be coupled to a precise knowledge of the immunological profile of the malady to be treated and the composition of the patients’ microbiome. Furthermore, we speculate that reduced efficacy of helminth therapy could be enhanced by combination with a probiotic matched to compensate for dysbiosis in a particular individual.

## Methods

### Mice and *H. diminuta* life-cycle

All experimental procedures were approved by the Univ. Calgary Animal Care Committee under protocol AC17-0115 in compliance with the Canadian Council on Animal Care guidelines.

Male BALB/c and C57BL/6 mice (7–9 weeks old, Charles River Laboratories, Quebec, Canada) were housed in HEPA filtered micro-isolator cages with free access to rodent chow (Pico-Vac Mouse Diet 20: 5062) and water in a 22 °C-controlled facility on a 12 h:12 h light:dark cycle. Breeding pairs of C57Bl/6 *Ffar2*^*+/−*^ mice were provided by Dr. B.T. Layden (University of Illinois, Chicago) [54] and maintained at the Univ. of Calgary. Germ-free (GF) BALB/c and C57BL/6 mice were bred and maintained in flexible-film sterile isolators in the International Microbiome Center at the Univ. Calgary. Germ-free status was tested by Sytox Green nucleic acid staining (Invitrogen) of caecal contents [25]. Mice were humanely euthanized prior to necropsy.

Adult *H. diminuta* were maintained in Sprague-Dawley Rats (Charles River) as a reservoir host and gravid proglottids passaged through flour beetles to obtain the infective cysticercoids. Mice, under mild manual restrain, were infected with five cysticercoids in 100 μL of 0.9% NaCl with a round-tipped oral gavage needle [6]. For GF mice, cysticercoids were incubated in antibiotics (300 μL: kanamycin (400 mg/L), gentamicin (35 mg/L), colistin (42 mg/L), and metronidazole (215 mg/L)) for 2 h at 37 °C. Cysticercoid viability after antibiotics treatment was confirmed by excystment in vitro and ability to infect *il4ra*^*−/−*^ mice [25]). Each GF-mouse received 8–10 cysticercoids by oral gavage.

### Induction and assessment of DNBS-colitis

Colitis was induced in anesthetized mice with 3 mg di-nitrobenzene sulphonic acid (DNBS: MP Biomedicals, Santa Ana, CA) in 100 μL of 50% ethanol in PBS via a polyethylene catheter inserted 3 cm into the colon [6]. Bodyweight was recorded daily over 72 h and on necropsy, colon length was measured and a macroscopic disease activity score (DAS) was calculated (maximum 5 points) [6]. Portions of mid-colon were excised, fixed in 10% neutral-buffered formalin, dehydrated, and embedded in paraffin wax. Seven μm sections were collected on coded slides, stained with hematoxylin and eosin, and histopathology scored in a blinded fashion on a validated 12-point scale [6]. Additional histological sections were immunostained for il-10 receptor-*α* chain using a detection rabbit-anti-mouse il-10 antibody (1:100 in PBS; Abcam ab225820). After 24 h 4^o^C incubation, sections were washed, secondary goat-anti-rabbit HRP-conjugated antibody (1:500 in PBS, 30 min room temperature) applied, washed, and then DAB (3,3′Diaminobenzidine) substrate (Abcam: ab64238) added. Representative images were captured on an Olympus BX41 microscope fitted with a U-TMAD T mount adapter, using cell Sens standard software (Olympus). Images were processed using ImageJ (version 1.80 https://imagej. nih.gov/ij/).

Approximately 0.5 cm of tissue immediately distal to that taken for histology was collected and total RNA isolated using the Aurum Total RNA Mini Kit (Bio-Rad Laboratories, Hercules, CA) as per the manufacturer’s protocol, quantified with the Nanodrop 1000 Spectrophotometer (Thermo Fisher Scientific, Wilmington, DE), and 0.5 μg of RNA was converted to cDNA using an iScript kit (Bio-Rad Lab). Quantitative real-time polymerase chain reaction (qPCR) of murine colonic tissue was performed as previously described [25, 55] using primer sequences shown in Suppl. Table 1.

Interleukin-10 production by concanavalin-A (2 μg/mL, 48 h)-stimulated spleen cells (5 × 10^6^/mL) was determined by sandwich ELISA using paired antibodies (R&D Systems Inc.) in accordance with the manufacturer’s instructions [25].

### Antibiotic treatment of mice

Mice were treated with a broad-spectrum cocktail of antibiotics (ABX: drinking water, kanamycin (40 mg/L), gentamicin (3.5 mg/L), colistin (4.2 mg/L), and metronidazole (21.5 mg/L) and ip. injections of vancomycin (200 μL of 0.5 mg/mL) [56] vancomycin only, or polymyxin B (PMB; 1 g/L) + neomycin sulfate (Neo; 500 mg/L) in drinking water (see figures for treatment regimens) [45].

### 16S rRNA analysis of bacterial communities

Feces (100 mg) was homogenized using 0.2 g of 2.8 mm ceramic beads (Mo Bio Laboratories, #13114-50) in a Bullet Blender (Next Advance) and DNA isolated following the method of Surette et al., [57] and bacterial community profiling performed via 16S rRNA V3-V4 region (341F-785F) amplicon sequencing via Illumina MiSeq (*25*). Analysis was performed using Rstudio (R version 3.5.0). Prior to processing the raw fastq files, adapter and primer sequences were removed using the Cutadapt program (version 1.17). Once non-biological nucleotides were removed, the paired-end fastq files were processed using the dada2 pipeline (version 1.12.1; dada2 workflow http://benjjneb.github.io/dada2/tutorial.html). Using the dada2 “filterAndTrim” function, the truncation lengths were set to 270 and 200 and the maximum number of expected errors was set at 2. After learning the error rates (“learnErrors” in dada2) for denoising the amplicon data of non-biological errors (“dada” in dada2), forward and reverse reads were merged for full-denoised sequences (“mergePairs” in dada2) and an amplicon sequence variant (ASV) table generated (“makeSequenceTable” in dada2). Taxonomic classifications were assigned to the ASV table (“assignTaxonomy” in dada2) using the Silva 132 database (arb-silva.de/documentation/release-132/) as a reference training set. Community analysis of the data was performed using Phyloseq version 1.24.2. Alpha diversity was determined using the “plot_richness” function in Phyloseq and Wilcoxon rank sum test assessed statistical significance. Using the “Unifrac” function in Phyloseq, weighted Unifrac distances of each sample was determined and plotted using Principal Coordinate Analysis (PCoA). A permanova test using the “Adonis” function (Vegan version 2.5-6.) tested for statistically significant compositional differences between groups (*β*-diversity). Following a positive permanova test, a permutation test for homogeneity of multivariate dispersions was performed, for which a non-significant test would indicate that the permanova test is a real result and not due to differences in group dispersion. Differential abundance (identifying taxa within a sample/group that are significantly increased or decreased when compared to another sample) was performed with R program DeSeq2 (v. 3.11). Data are displayed as a log-fold change. The raw fastq sequencing files used within this study have been uploaded to the short reads archive (SRA) database (BioProjectID:PRJNA690571).

### Short-chain fatty acid and metabolite measurement

Five hundred milligrams of fresh feces was mixed with 500 μL 100% HPLC grade methanol and 500 μL of HPLC grade H_2_O, vortexed and then centrifuged at 13,000×*g* for 5 min at 4 °C. Then 700 μL of the supernatant was mixed with an equal volume of 50% HLPC grade methanol, vortexed (2 min), and spun down (13,000×*g*, 10 min, 4 °C). The supernatant was collected, divided into two, filtered (0.2 μm), and dried at 4 °C. One of the duplicate dried samples was reconstituted in 800 μL of deuterium oxide, titrated to a pH of 7.400 ± 0.005, and subjected to nuclear magnetic resonance (NMR) analysis [58]. NMR data were acquired on a 600 MHz Bruker Advance III instrument. Metabolites were assigned by ^1^H-^13^C heteronuclear single quantum coherence (HSQC). Data were collected using the hsqcetgpsp (Bruker) pulse program. Spectra were acquired in 8190 points in a 12.01 ppm sweep width in the direct dimension and 1024 increments, 110 ppm sweep width in the indirect dimension. Data were processed in Burker TopSpin and analyzed in rNMR. Metabolites were assigned using the Madison Metabolomics Consortium Database reference spectra available from the BMRB. Once the spectra had been assigned, metabolites were quantified using 1D ^1^H NMR with NOSEY water suppression (Bruker noesygppr1d pulse program). Data were acquired in 65,536 points with 32 scans and a sweep width of 12.01 ppm. Metabolites were quantified following established methods [58].

In other experiments, 100 mg of feces was assessed by liquid chromatography-mass spectrometry (LC-MS) for SCFA [59]. Samples were dissolved in ice-cold extraction solvent containing 100 μL of H_2_O/acetonitrile (50:50) solution containing 5 mM, 200 μM and 500 μM of ^13^C-labeled acetic acid (1,2-^13^C2, 99 atom%: #CLM-113, Cambridge Isotope Lab.) propionic acid (99 atom %: #589586, Sigma-Aldrich) and butyric acid (-1,2-^13^C2 99 atom % ^13^C: #491993, Sigma-Aldrich), respectively [for fecal samples from antibiotic-treated mice the internal SCFA standards were 2.5 mM, 200 μM and 50 μM, respectively]. Samples were vortexed, then centrifuged (10,000×*g*, 10 min, 4 °C), and when clear 50 μL of supernatant was cooled to 0 °C and derivatized in an extraction solvent containing 2.5 μL of 2.4 M aniline (dissolved in acetonitrile), followed by 2.5 μL of 1.2 M 1-ethyl-3(3-dimethylaminopropyl) carbodiimide (dissolved in H_2_O). The mixture was vortexed for 15 s and placed on ice for 2 h (vortexing every 30 min), diluted 1:20 with 50% methanol, vortexed for 15 s, and the samples subjected LC-MS analysis. Data were analyzed as previously described [60]. Briefly, metabolites were separated on a reverse phase chromatographic gradient (Thermo Fisher Hypersil GOLD TMC18 column) and metabolites were quantified by selected reaction monitoring (SRM). Concentrations were calculated based on the ratio of isotope-labeled fragments from standard compounds relative to the corresponding fragments from microbial metabolites.

### Fecal microbial transplants (FMT) and bacteria-free fecal filtrates

Feces were collected from control male C57BL/6 mice or those infected with *H. diminuta* 8 days previously and immediately placed in 10 mL of pre-reduced sterile PBS in a Ruskinn anaerobic chamber. Samples were vortexed (2 min), centrifuged (5 min at 1000×*g*) and 400 μL of the fecal supernatant was given to GF-mice by oral gavage. Four weeks later, fecal samples were collected for 16S rRNA analysis, and mice were challenged with DNBS (5 mg in 100 μL 50% etoh.). In other experiments, fecal samples (500 mg) were collected from control mice and those infected with *H. diminuta* 8 days previously, solubilized in 10 mL of sterile PBS and passed through a 0.45 μm and then 0.2 μm pore-size filter [61]. This sterile filtrate was then administered as a 200 μL of 50 mg/mL enema to naïve specific pathogen-free (SPF) mice, that subsequently received DNBS.

### Treatment of mice with short-chain fatty acids (SCFAs)

Adopting published methodologies for treatment with SCFAs, male BALB/c mice were supplemented with 200 mM of sodium acetate (Sigma-Aldrich #S2889) [62] in their drinking water 7 days prior to DNBS and maintained on sodium acetate-drinking water throughout the experiment. Another cohort of animals received butyrate enemas (500 μL of 100 mM 98% sodium butyrate; Sigma-Aldrich #B5887) [44] or PBS (500 μL) 24 h and 3 h before DNBS and again at 24 h and 48 h post-DNBS administration.

### Anti-IL-10 antibody treatment of mice

Following a protocol we applied previously [6], mice received intraperitoneal injections of either a neutralizing anti-il-10 antibody (clone JES5-2A5; Biolegend #504909) or an isotype matched irrelevant IgG_1_ (Biolegend #400432) at day-3 (50 μg), day-7 (100 μg), and day-9 (50 μg) post-infection with *H. diminuta* for a total of 200 μg of antibody. DNBS was administered at 8 days post-infection and mice were necropsied 3 days later.

### Data presentation and statistical analysis

Results are expressed as the mean ± standard error of the mean (SE) and *n* is the number of mice. Data are analyzed using Graph Pad Prism 8.0 in which statistical comparisons for parametric data were performed via one-way ANOVA with Tukey’s post-test and the Kruskal-Wallis test with Dunn’s post-test was applied to non-parametric data. *P* < 0.05 was set as the level of acceptable statistical difference.

## Supplementary Information

**Additional file 1: Suppl. Figure 1.** Representative images of H&E stained sections of mid-colon from mice treated with broad-spectrum and DNBS. **Suppl. Figure 2.** Vancomycin or a combination of polymyxin B + neomycin impact gut bacteria in *H. diminuta* ± DNBS-treated mice. **Suppl. Figure 3.** Treatment with selected antibiotics interferes with *H. diminuta*-evoked inhibition of colitis. **Suppl. Figure 4.** Treatment with selected antibiotics affects stimulated splenic il-10 output. **Suppl. Figure 5.** *H. diminuta*-infected germ-free (GF) mice are not protected from DNBS-induced colitis. **Suppl. Figure 6.** Germ-free (GF) mice colonized with feces from *H. diminuta*-infected retain a distinct bacterial composition. **Suppl. Figure 7.** Short-chain fatty acids (SCFA) suppress DNBS-induced colitis. **Suppl. Figure 8.** Neutralizing anti-il-10 antibody block *H. diminuta*-evoked inhibition of DNBS-induced colitis.

**Additional file 2: Suppl. Table 1.** Primer sequences used throughout this study.

## Data Availability

The raw fastq sequencing files used within this study have been uploaded to the short reads archive (SRA) database (BioProjectID:PRJNA690571).

## References

[1] Ng SC (2018). Worldwide incidence and prevalence of inflammatory bowel disease in the 21st century: a systematic review of population-based studies. Lancet.

[2] Bach J-F (2018). The hygiene hypothesis in autoimmunity: the role of pathogens and commensals. Nat Rev Immunol.

[3] Helmby H (2015). Human helminth therapy to treat inflammatory disorders - where do we stand?. BMC Immunol.

[4] Ryan SM, Eichenberger RM, Ruscher R, Giacomin PR, Loukas A (2020). Harnessing helminth-driven immunoregulation in the search for novel therapeutic modalities. PLoS pathogens.

[5] Sorobetea D, Svensson-Frej M, Grencis R (2018). Immunity to gastrointestinal nematode infections. Mucosal Immunol.

[6] Hunter MM, Wang A, Hirota CL, McKay DM (2005). Neutralizing anti-IL-10 antibody blocks the protective effect of tapeworm infection in a murine model of chemically induced colitis. J Immunol.

[7] Smith P, Mangan NE, Walsh CM, Fallon RE, McKenzie ANJ, van Rooijen N, Fallon PG (2007). Infection with a helminth parasite prevents experimental colitis via a macrophage-mediated mechanism. J Immunol.

[8] Mishra PK, Patel N, Wu W, Bleich D, Gause WC (2013). Prevention of type 1 diabetes through infection with an intestinal nematode parasite requires IL-10 in the absence of a Th2-type response. Mucosal Immunol.

[9] McSorley HJ, Blair NF, Robertson E, Maizels RM (2015). Suppression of OVA-alum induced allergy by Heligmosomoides polygyrus products is MyD88-, TRIF-, regulatory T- and B cell-independent, but is associated with reduced innate lymphoid cell activation. Exp Parasitol.

[10] Terrazas C, de Dios Ruiz-Rosado J, Amici SA, Jablonski KA, Martinez-Saucedo D, Webb LM, Cortado H, Robledo-Avila F, Oghumu S, Satoskar AR, Rodriguez-Sosa M, Terrazas LI, Guerau-de-Arellano M, Partida-Sánchez S (2017). Helminth-induced Ly6Chi monocyte-derived alternatively activated macrophages suppress experimental autoimmune encephalomyelitis. Scientific reports.

[11] Cheng Y, Zhu X, Wang X, Zhuang Q, Huyan X, Sun X, Huang J, Zhan B, Zhu X (2018). Trichinella spiralis infection mitigates collagen-induced arthritis via programmed death 1-mediated immunomodulation. Front Immunol.

[12] Espinoza-Jimenez A, De Haro R, Terrazas LI (2017). Taenia crassiceps antigens control experimental type 1 diabetes by inducing alternatively activated macrophages. Mediators Inflamm.

[13] Reyes JL, Wang A, Fernando MR, Graepel R, Leung G, van Rooijen N, Sigvardsson M, McKay DM (2015). Splenic B cells from Hymenolepis diminuta-infected mice ameliorate colitis independent of T cells and via cooperation with macrophages. J Immunol.

[14] Li Y, Guan X, Liu W, Chen HL, Truscott J, Beyatli S, Metwali A, Weiner GJ, Zavazava N, Blumberg RS, Urban JF, Blazar BR, Elliott DE, Ince MN (2018). Helminth-induced production of TGF-β and suppression of graft-versus-host disease is dependent on IL-4 production by host cells. J Immunol.

[15] Kitagaki K, Businga TR, Racila D, Elliott DE, Weinstock JV, Kline JN (2006). Intestinal helminths protect in a murine model of asthma. J Immunol.

[16] Hang L, Kumar S, Blum AM, Urban JF, Fantini MC, Weinstock JV (2019). Heligmosomoides polygyrus bakeri infection decreases Smad7 expression in intestinal CD4^+^ T cells, which allows TGF-β to induce IL-10-producing regulatory T cells that block colitis. J Immunol.

[17] Ramanan D, Bowcutt R, Lee SC, Tang MS, Kurtz ZD, Ding Y, Honda K, Gause WC, Blaser MJ, Bonneau RA, Lim YAL, Loke P’, Cadwell K (2016). Helminth infection promotes colonization resistance via type 2 immunity. Science.

[18] Reynolds LA, Finlay BB, Maizels RM (2015). Cohabitation in the intestine: interactions among helminth parasites, bacterial microbiota, and host immunity. J Immunol.

[19] Lee SC, Tang MS, Lim YAL, Choy SH, Kurtz ZD, Cox LM, Gundra UM, Cho I, Bonneau R, Blaser MJ, Chua KH, Loke P' (2014). Helminth colonization is associated with increased diversity of the gut microbiota. PLoS Negl Trop Dis.

[20] Kumar NP, Kathamuthu GR, Moideen K, Banurekha VV, Nair D, Fay MP, Nutman TB, Babu S (2020). Strongyloides stercoralis coinfection is associated with greater disease severity, higher bacterial burden and elevated plasma matrix metalloproteinases in pulmonary tuberculosis. J Infect Dis.

[21] Ducarmon QR (2020). Dynamics of the bacterial gut microbiota during controlled human infection with Necator americanus larvae. Gut Microbes.

[22] Zaiss MM, Rapin A, Lebon L, Dubey LK, Mosconi I, Sarter K, Piersigilli A, Menin L, Walker AW, Rougemont J, Paerewijck O, Geldhof P, McCoy KD, Macpherson AJ, Croese J, Giacomin PR, Loukas A, Junt T, Marsland BJ, Harris NL (2015). The intestinal microbiota contributes to the ability of helminths to modulate allergic inflammation. Immunity.

[23] Rapin A, Chuat A, Lebon L, Zaiss MM, Marsland BJ, Harris NL (2020). Infection with a small intestinal helminth, Heligmosomoides polygyrus bakeri, consistently alters microbial communities throughout the murine small and large intestine. Internat J Parasitol.

[24] McKay DM (2010). The immune response to and immunomodulation by Hymenolepis diminuta. Parasitology.

[25] Shute A, Wang A, Jayme TS, Strous M, McCoy KD, Buret AG, McKay DM (2020). Worm expulsion is independent of alterations in composition of the colonic bacteria that occur during experimental Hymenolepis diminuta-infection in mice. Gut Microbes.

[26] Zheng L, Kelly CJ, Battista KD, Schaefer R, Lanis JM, Alexeev EE, Wang RX, Onyiah JC, Kominsky DJ, Colgan SP (2017). Microbial-derived butyrate promotes epithelial barrier function through IL-10 receptor-dependent repression of claudin-2. J Immunol.

[27] Lopes F, Matisz C, Reyes JL, Jijon H, al-Darmaki A, Kaplan GG, McKay DM (2016). Helminth regulation of immunity: a three-pronged approach to treat colitis. Inflamm Bowel Dis.

[28] Summers RW, Elliott DE, Urban JF, Thompson RA, Weinstock JV (2005). Trichuris suis therapy for active ulcerative colitis: a randomized controlled trial. Gastroenterology.

[29] Summers RW, Elliott DE, Urban JF, Thompson R, Weinstock JV (2005). Trichuris suis therapy in Crohn's disease. Gut.

[30] Correale J, Farez MF (2011). The impact of parasite infections on the course of multiple sclerosis. J Neuroimmunol.

[31] Fleming JO, Isaak A, Lee JE, Luzzio CC, Carrithers MD, Cook TD, Field AS, Boland J, Fabry Z (2011). Probiotic helminth administration in relapsing-remitting multiple sclerosis: a phase 1 study. Mult Scler.

[32] Fleming J (2017). Safety and efficacy of helminth treatment in relapsing-remitting multiple sclerosis: Results of the HINT 2 clinical trial. Mult Scler.

[33] Scholmerich J (2017). A randomised, double-blind, placebo-controlled trial of Trichuris suis ova in active Crohn's disease. J Crohn's & Colitis.

[34] Huang X, Zeng LR, Chen FS, Zhu JP, Zhu MH (2018). Trichuris suis ova therapy in inflammatory bowel disease: A meta-analysis. Medicine.

[35] Lloyd-Price J (2019). Multi-omics of the gut microbial ecosystem in inflammatory bowel diseases. Nature.

[36] Khan WI, Blennerhasset PA, Varghese AK, Chowdhury SK, Omsted P, Deng Y, Collins SM (2002). Intestinal nematode infection ameliorates experimental colitis in mice. Infect Immun.

[37] Ramanan D, Tang MS, Bowcutt R, Loke P, Cadwell K (2014). Bacterial sensor Nod2 prevents inflammation of the small intestine by restricting the expansion of the commensal Bacteroides vulgatus. Immunity.

[38] Floudas A (2019). Schistosoma mansoni worm infection regulates the intestinal microbiota and susceptibility to colitis. Infect Immun.

[39] Jirků M (2021). Helminth interactions with bacteria in the host gut are essential for its immunomodulatory effect. Microorganisms.

[40] Ryan FJ, Ahern AM, Fitzgerald RS, Laserna-Mendieta EJ, Power EM, Clooney AG, O’Donoghue KW, McMurdie PJ, Iwai S, Crits-Christoph A, Sheehan D, Moran C, Flemer B, Zomer AL, Fanning A, O’Callaghan J, Walton J, Temko A, Stack W, Jackson L, Joyce SA, Melgar S, DeSantis TZ, Bell JT, Shanahan F, Claesson MJ (2020). Colonic microbiota is associated with inflammation and host epigenomic alterations in inflammatory bowel disease. Nat Commun.

[41] Shimokawa C (2019). Suppression of obesity by an intestinal helminth through interactions with intestinal microbiota. Infect Immun.

[42] Kong L, Lloyd-Price J, Vatanen T, Seksik P, Beaugerie L, Simon T, Vlamakis H, Sokol H, Xavier RJ (2020). Linking strain engraftment in fecal microbiota transplantation with maintenance of remission in Crohn's disease. Gastroenterology.

[43] Vernia P, Annese V, Bresci G, D’Albasio G, D’Incà R, Giaccari S, Ingrosso M, Mansi C, Riegler G, Valpiani D, Caprilli R, GISC (Gruppo Italiano per lo Studio del Colon and del Retto) (2003). Topical butyrate improves efficacy of 5-ASA in refractory distal ulcerative colitis: results of a multicentre trial. Eur J Clin Invest.

[44] Butzner JD, Parmar R, Bell CJ, Dalal V (1996). Butyrate enema therapy stimulates mucosal repair in experimental colitis in the rat. Gut.

[45] Maslowski KM, Vieira AT, Ng A, Kranich J, Sierro F, di Yu, Schilter HC, Rolph MS, Mackay F, Artis D, Xavier RJ, Teixeira MM, Mackay CR (2009). Regulation of inflammatory responses by gut microbiota and chemoattractant receptor GPR43. Nature.

[46] Chen G, Ran X, Li B, Li Y, He D, Huang B, Fu S, Liu J, Wang W (2018). Sodium butyrate inhibits inflammation and maintains epithelium barrier integrity in a TNBS-induced inflammatory bowel disease mice model. EBioMedicine.

[47] Chun E, Lavoie S, Fonseca-Pereira D, Bae S, Michaud M, Hoveyda HR, Fraser GL, Gallini Comeau CA, Glickman JN, Fuller MH, Layden BT, Garrett WS (2019). Metabolite-sensing receptor Ffar2 regulates colonic group 3 innate lymphoid cells and gut immunity. Immunity.

[48] Couto MR, Gonçalves P, Magro F, Martel F (2020). Microbiota-derived butyrate regulates intestinal inflammation: focus on inflammatory bowel disease. Pharmacol Res.

[49] Saraiva M, Vieira P, O'Garra A (2020). Biology and therapeutic potential of interleukin-10. J Exp Med.

[50] Su CW, Chen CY, Jiao L, Long SR, Mao T, Ji Q, O’Donnell S, Stanton C, Zheng S, Walker WA, Cherayil BJ, Shi HN (2020). Helminth-induced and Th2-dependent alterations of the gut microbiota attenuate obesity caused by high-fat diet. Cell Mol Gastroenterol Hepatol.

[51] Machiels K, Joossens M, Sabino J, de Preter V, Arijs I, Eeckhaut V, Ballet V, Claes K, van Immerseel F, Verbeke K, Ferrante M, Verhaegen J, Rutgeerts P, Vermeire S (2014). A decrease of the butyrate-producing species Roseburia hominis and Faecalibacterium prausnitzii defines dysbiosis in patients with ulcerative colitis. Gut.

[52] Thibault R, Blachier F, Darcy-Vrillon B, de Coppet P, Bourreille A, Segain JP (2010). Butyrate utilization by the colonic mucosa in inflammatory bowel diseases: a transport deficiency. Inflamm Bowel Dis.

[53] Shouval DS, Biswas A, Goettel JA, McCann K, Conaway E, Redhu NS, Mascanfroni ID, al Adham Z, Lavoie S, Ibourk M, Nguyen DD, Samsom JN, Escher JC, Somech R, Weiss B, Beier R, Conklin LS, Ebens CL, Santos FGMS, Ferreira AR, Sherlock M, Bhan AK, Müller W, Mora JR, Quintana FJ, Klein C, Muise AM, Horwitz BH, Snapper SB (2014). Interleukin-10 receptor signaling in innate immune cells regulates mucosal immune tolerance and anti-inflammatory macrophage function. Immunity.

[54] Fuller M, Priyadarshini M, Gibbons SM, Angueira AR, Brodsky M, Hayes MG, Kovatcheva-Datchary P, Bäckhed F, Gilbert JA, Lowe WL, Layden BT (2015). The short-chain fatty acid receptor, FFA2, contributes to gestational glucose homeostasis. Am J Physiol Endocrinol Metab.

[55] Jayme TS (2020). Human interleukin-4-treated regulatory macrophages promote epithelial wound healing and reduce colitis in a mouse model. Sci Adv.

[56] Lopes F, Wang A, Smyth D, Reyes JL, Doering A, Schenck LP, Beck P, Waterhouse C, McKay DM (2015). The Src kinase Fyn is protective in acute chemical-induced colitis and promotes recovery from disease. J Leukoc Biol.

[57] Heirali A, McKeon S, Purighalla S, Storey DG, Rossi L, Costilhes G, Drews SJ, Rabin HR, Surette MG, Parkins MD (2016). Assessment of the microbial constituents of the home environment of individuals with cystic fibrosis (CF) and their association with lower airways infections. PloS One.

[58] Lewis I (2007). Method for determining molar concentrations of metabolites in complex solutions from two-mimensional ^1^H−^13^C NMR spectra. Anal Chem.

[59] Wyss M, Brown K, Thomson CA, Koegler M, Terra F, Fan V, Ronchi F, Bihan D, Lewis I, Geuking MB, McCoy KD (2020). Using precisely defined in vivo microbiotas to understand microbial regulation of IgE. Front Immunol.

[60] Bihan D, Rydzak T, Wyss M, Pittman K, McCoy KD, Lewis IA. Method for absolute quantification of short chain fatty acids via reverse phase chromatography mass spectrometry. ChemRxiv. 2019;2019. 10.26434/chemrxiv.9955553.v1. 10.1371/journal.pone.0267093 PMC9020710 35443015

[61] Ott SJ, Waetzig GH, Rehman A, Moltzau-Anderson J, Bharti R, Grasis JA, Cassidy L, Tholey A, Fickenscher H, Seegert D, Rosenstiel P, Schreiber S (2017). Efficacy of sterile fecal filtrate transfer for treating patients with Clostridium difficile infection. Gastroenterology.

[62] Macia L, Tan J, Vieira AT, Leach K, Stanley D, Luong S, Maruya M, Ian McKenzie C, Hijikata A, Wong C, Binge L, Thorburn AN, Chevalier N, Ang C, Marino E, Robert R, Offermanns S, Teixeira MM, Moore RJ, Flavell RA, Fagarasan S, Mackay CR (2015). Metabolite-sensing receptors GPR43 and GPR109A facilitate dietary fibre-induced gut homeostasis through regulation of the inflammasome. Nat Commun.

