# Supplementary material for: Cooperation between host immunity and the gut bacteria is essential for helminth-evoked suppression of colitis
Source: Microbiome. 2021 Sep 13;9:186. doi: 10.1186/s40168-021-01146-2 (PMC8438845; doi:10.1186/s40168-021-01146-2)

Shute et al 2021, Supplementary Figure 1

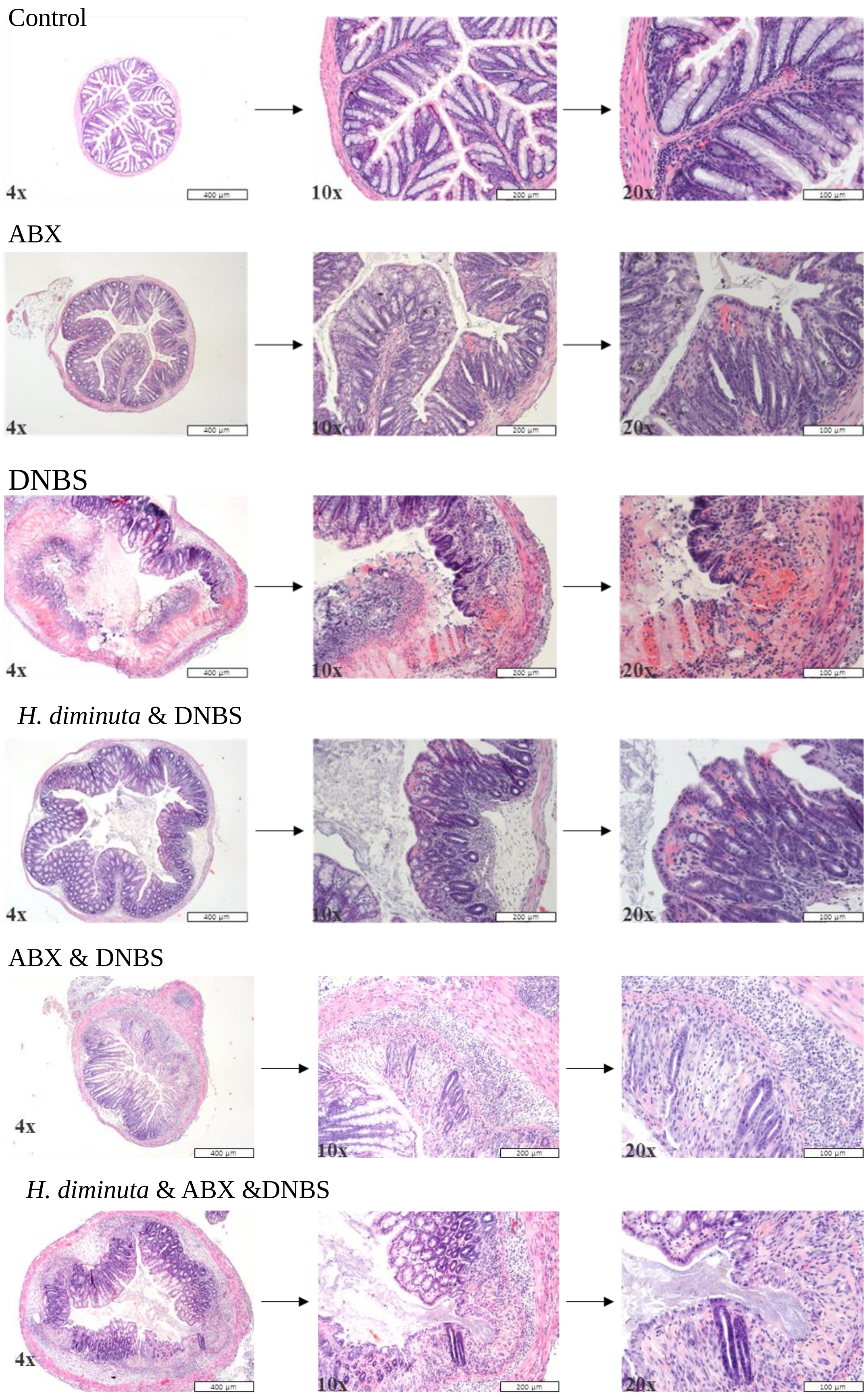

Shute et al 2021, Supplementary Figure 2A-C

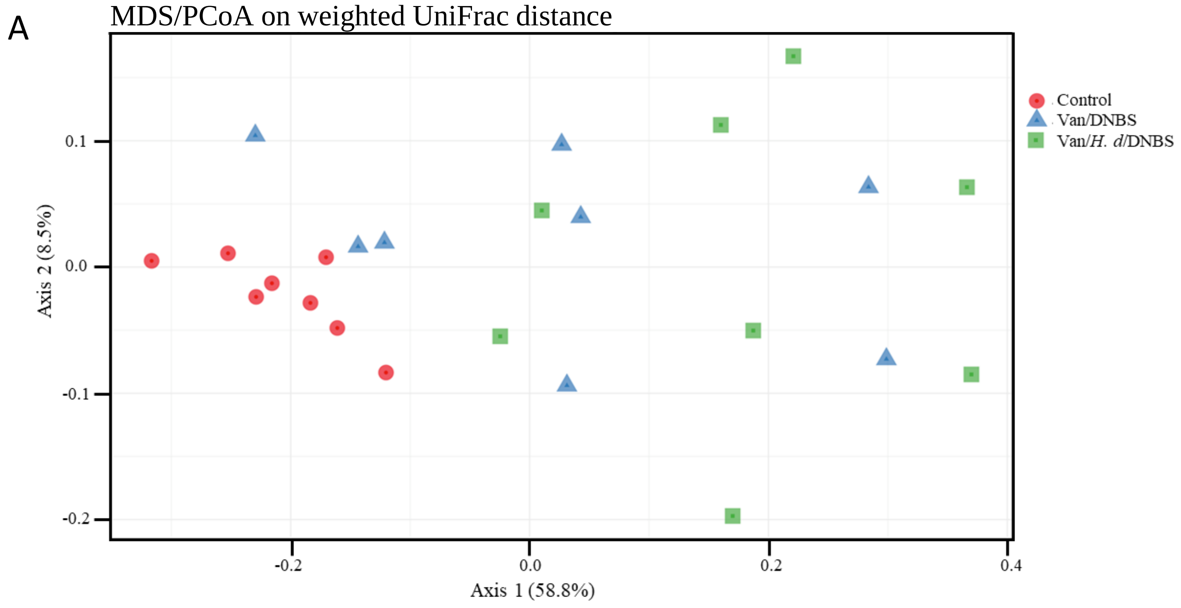

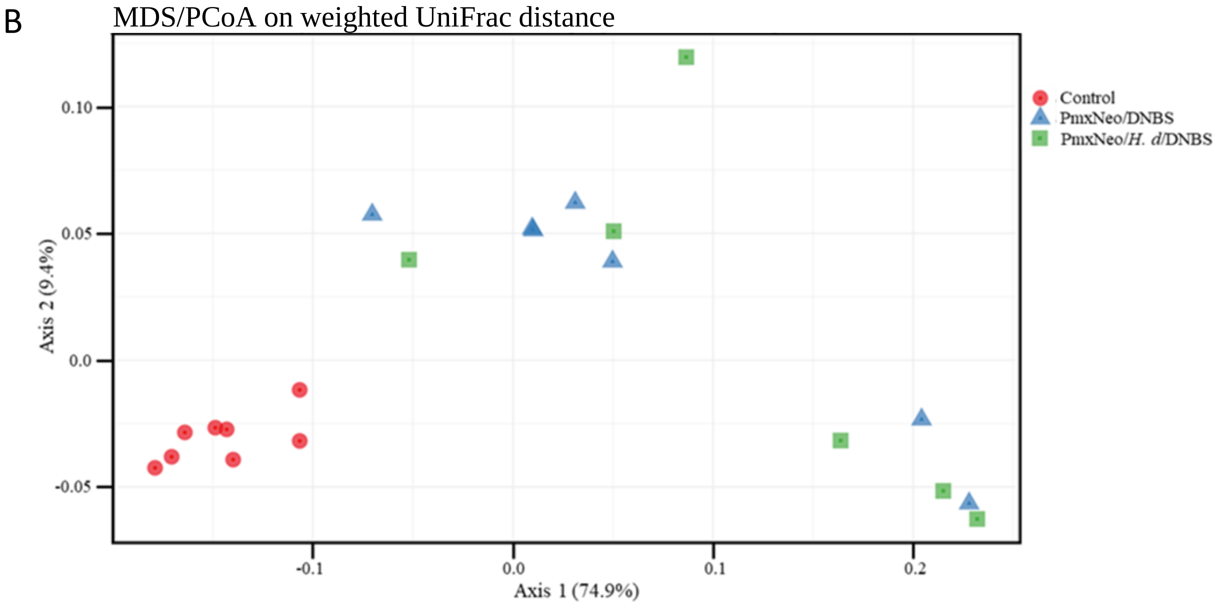

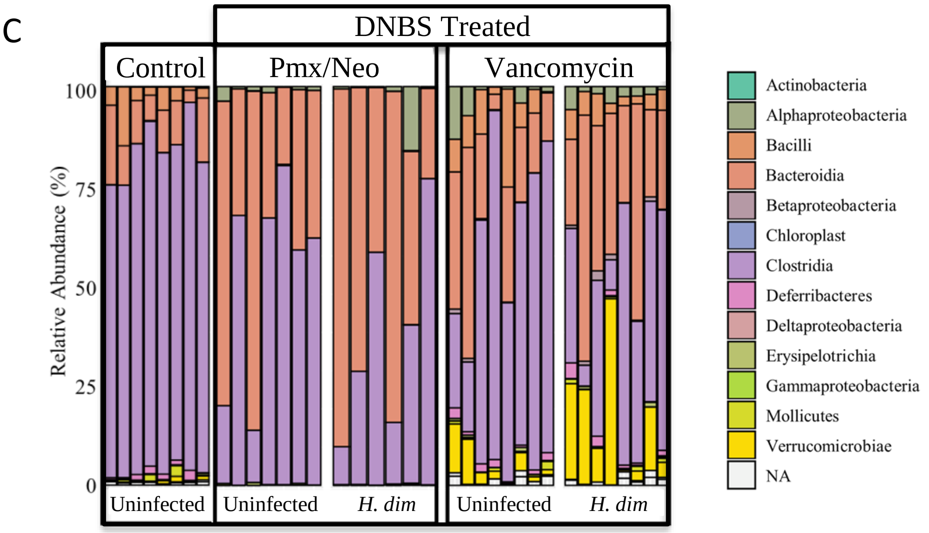

Shute et al 2021, Supplementary Figure 2D-E

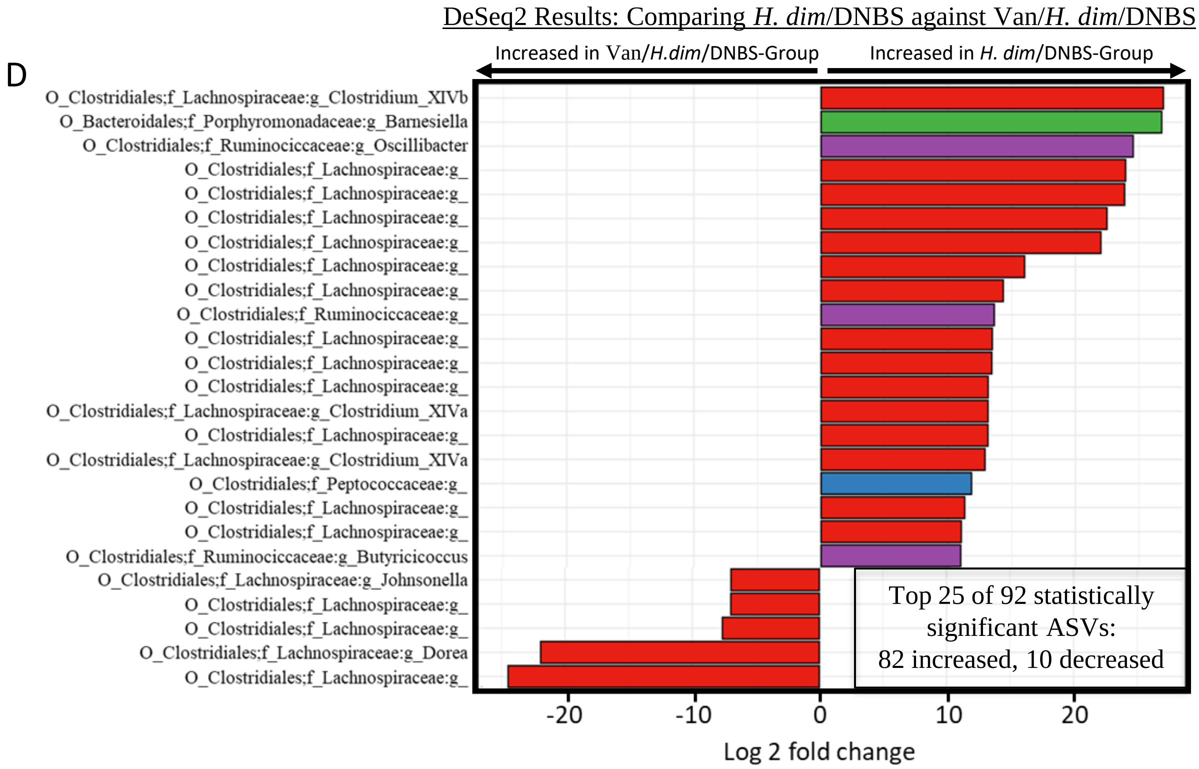

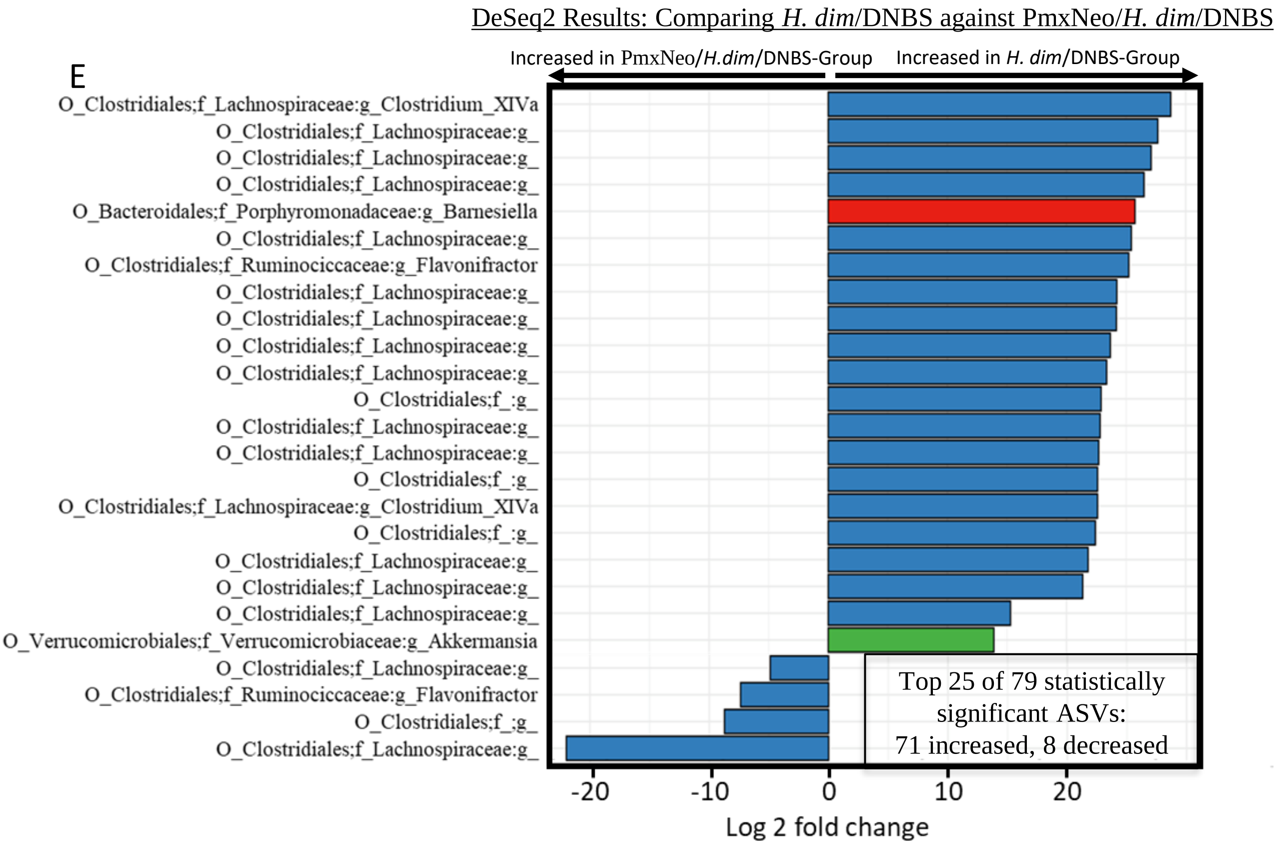

Shute et al 2021, Supplementary Figure 3

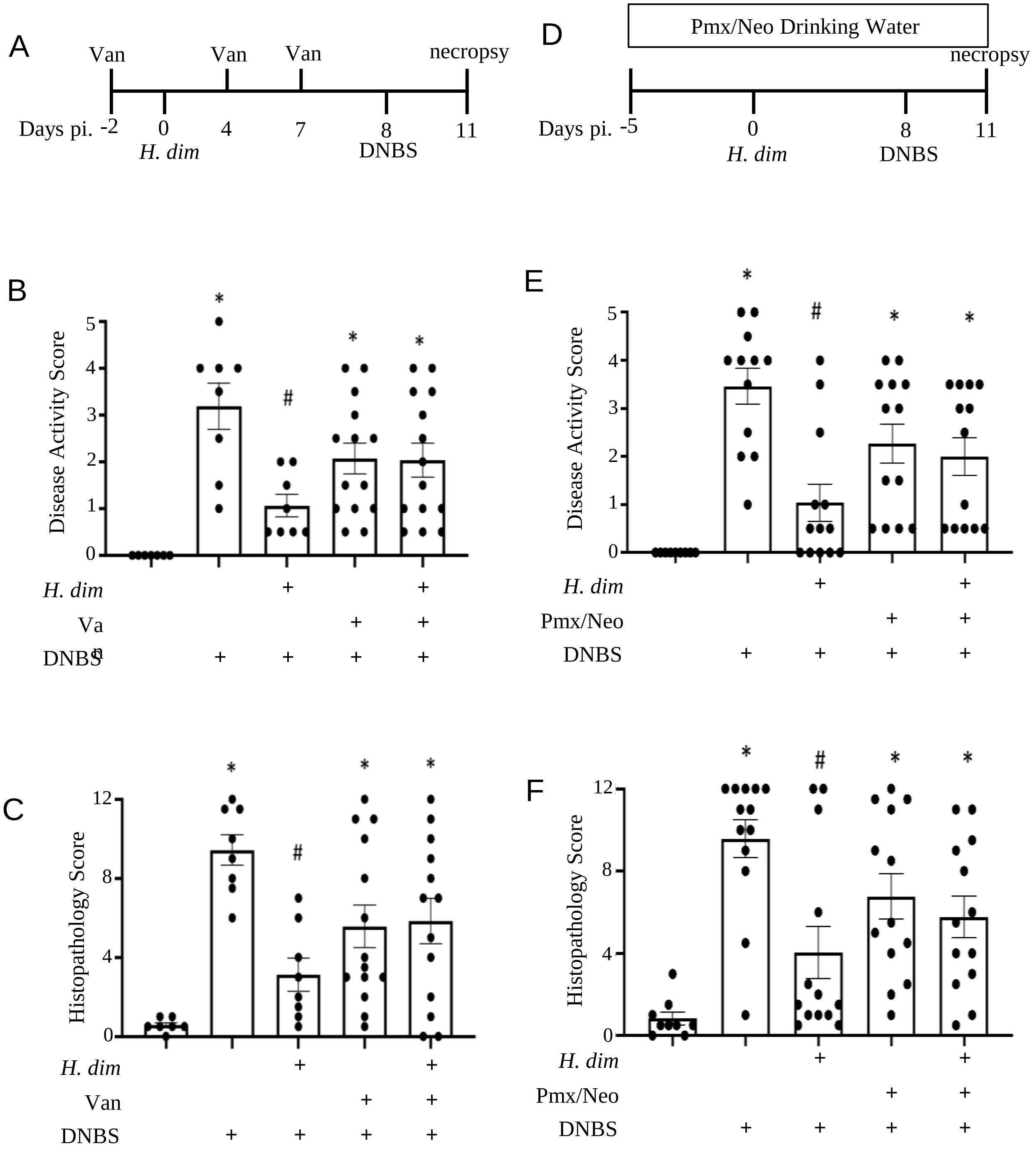

Shute et al 2021, Supplementary Figure 4

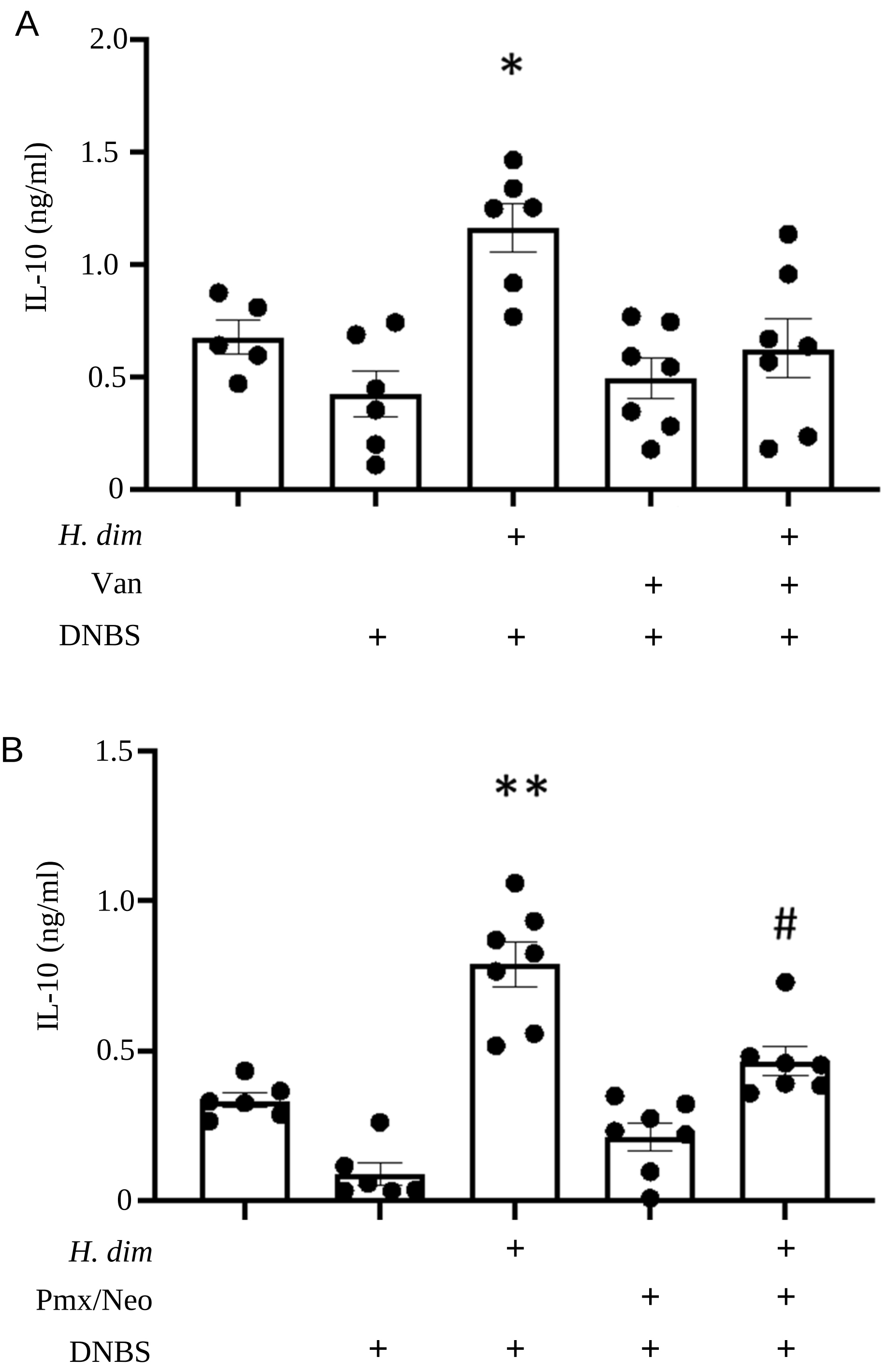

Shute et al 2010, Supplementary Figure 5

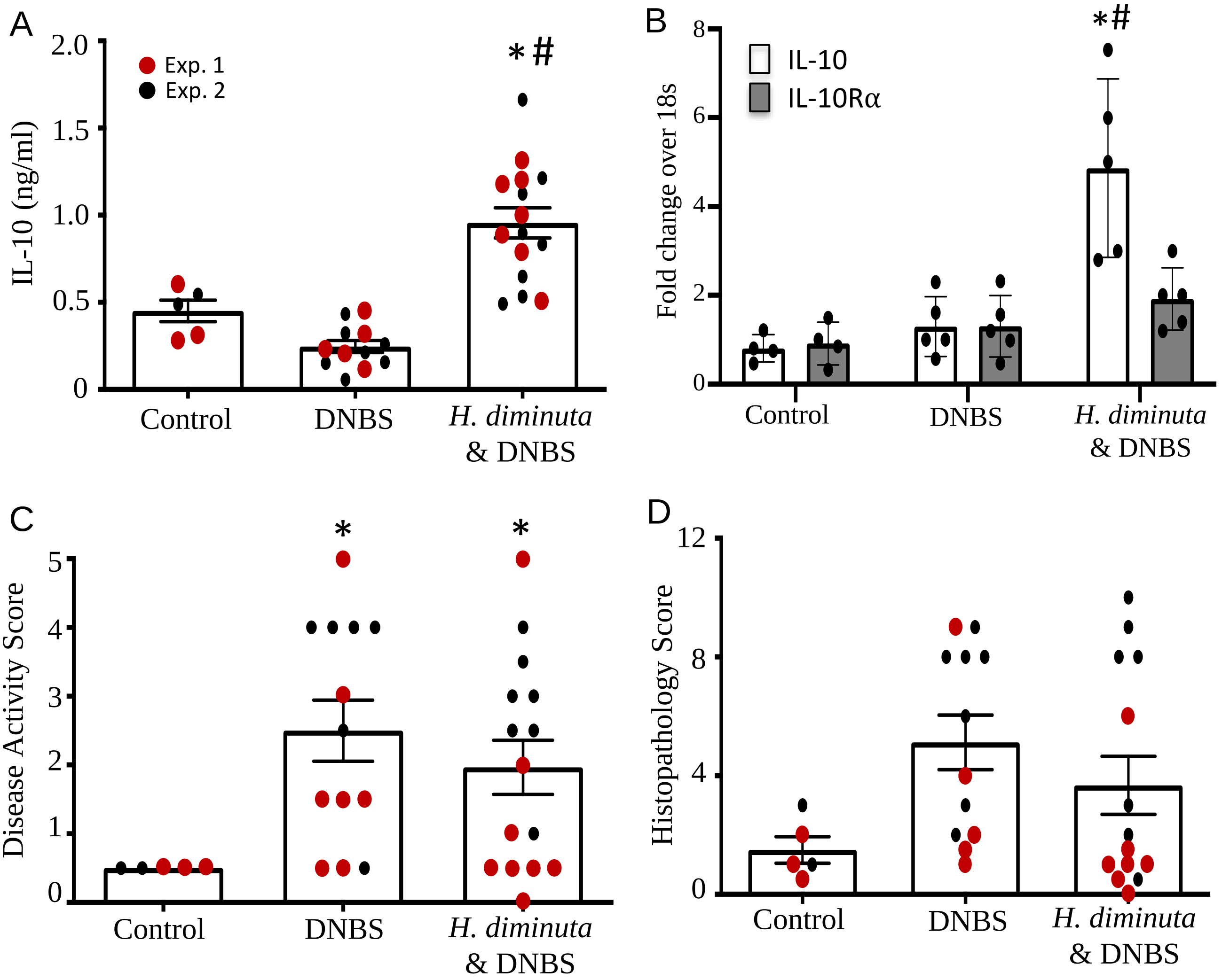

Shute et al 2021, Supplementary Figure 6A, B

A

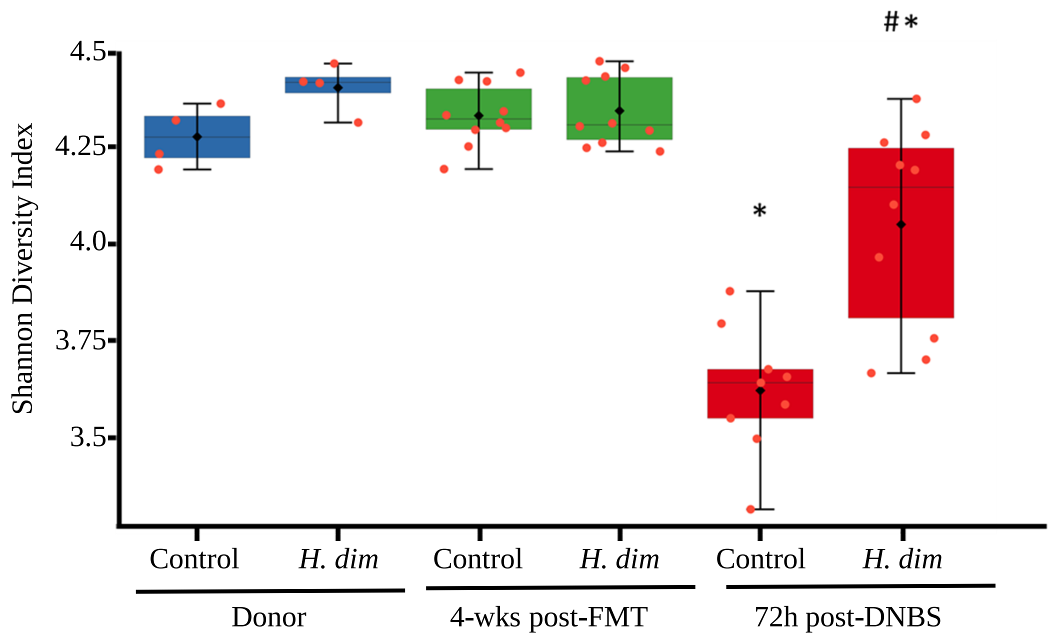

B

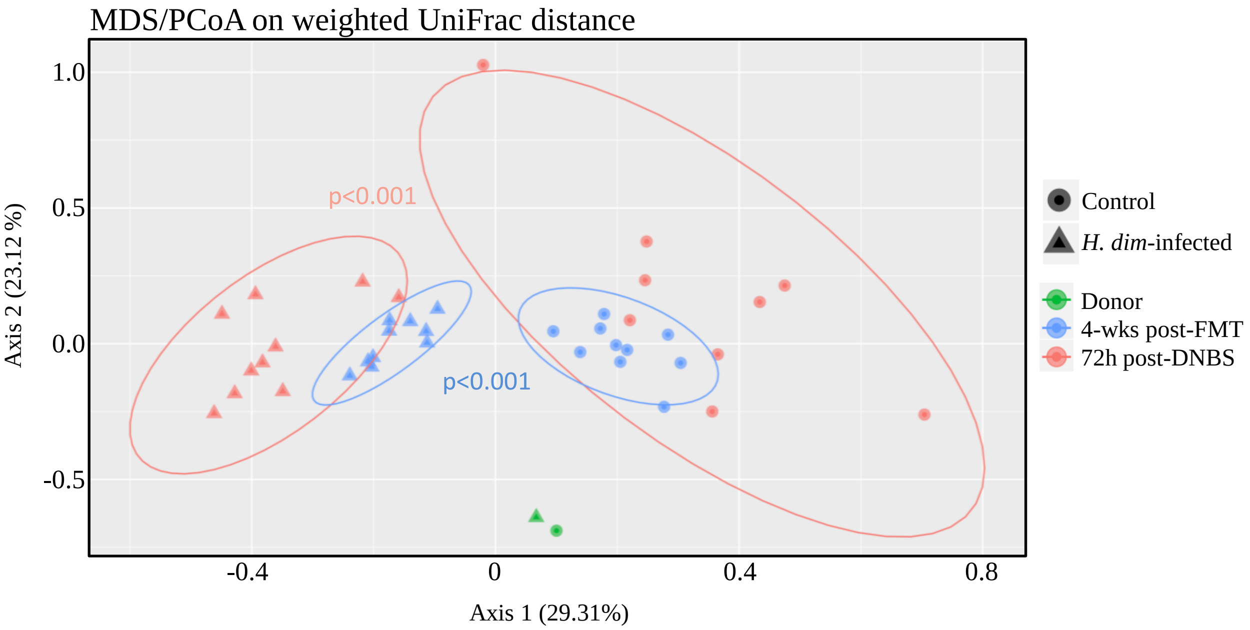

Shute et al 2021, Supplementary Figure 6C, D

C

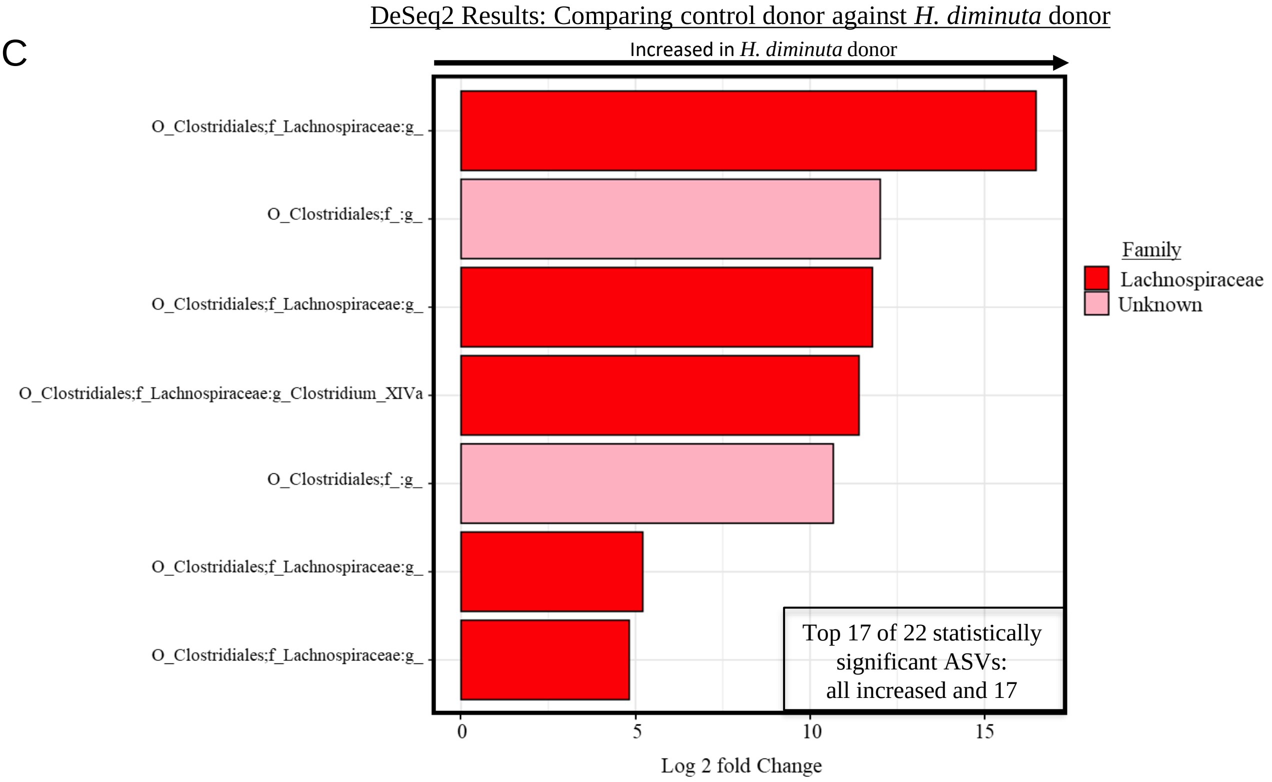

D

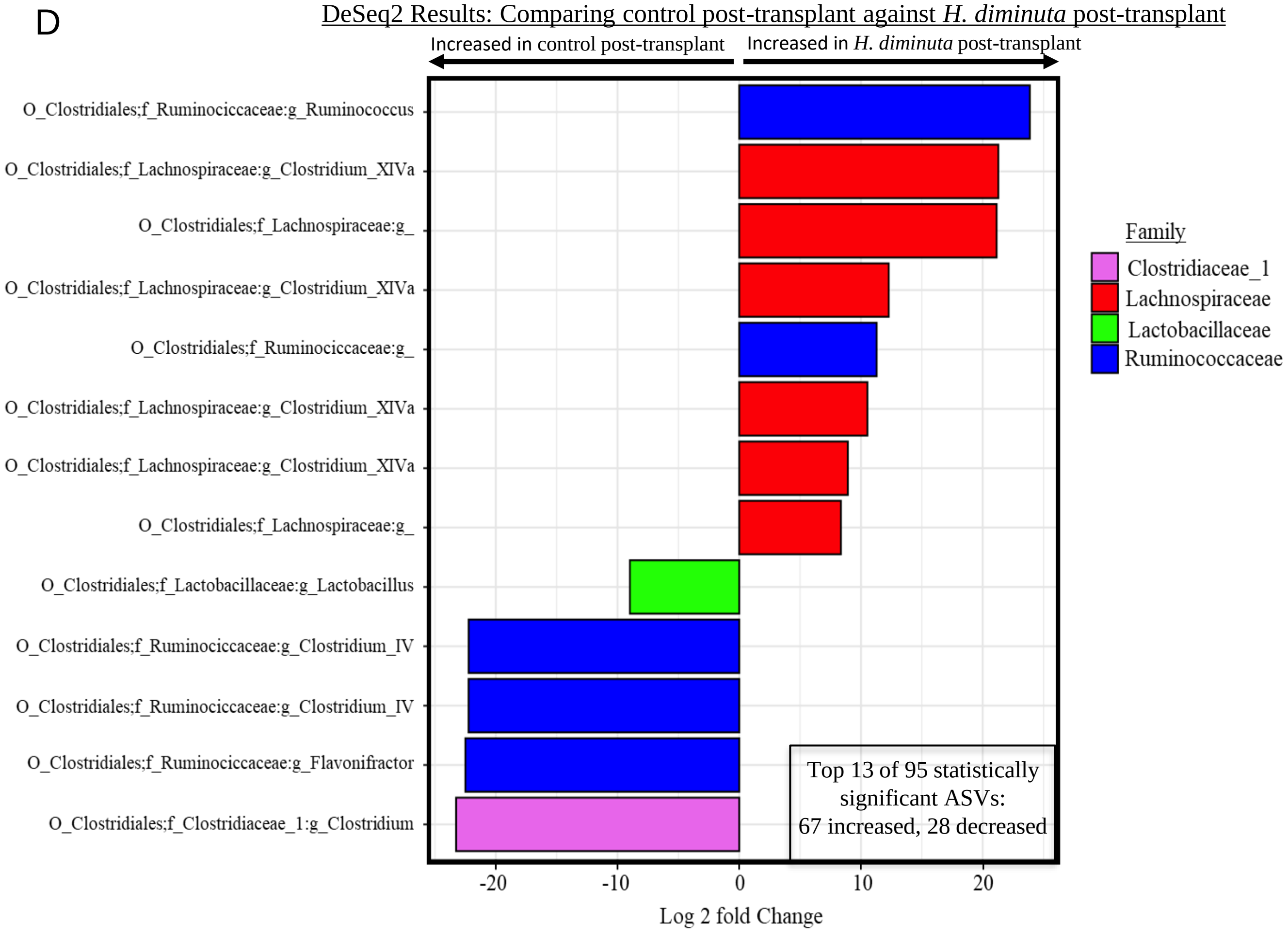

Shute et al 2021, Supplementary Figure 6E

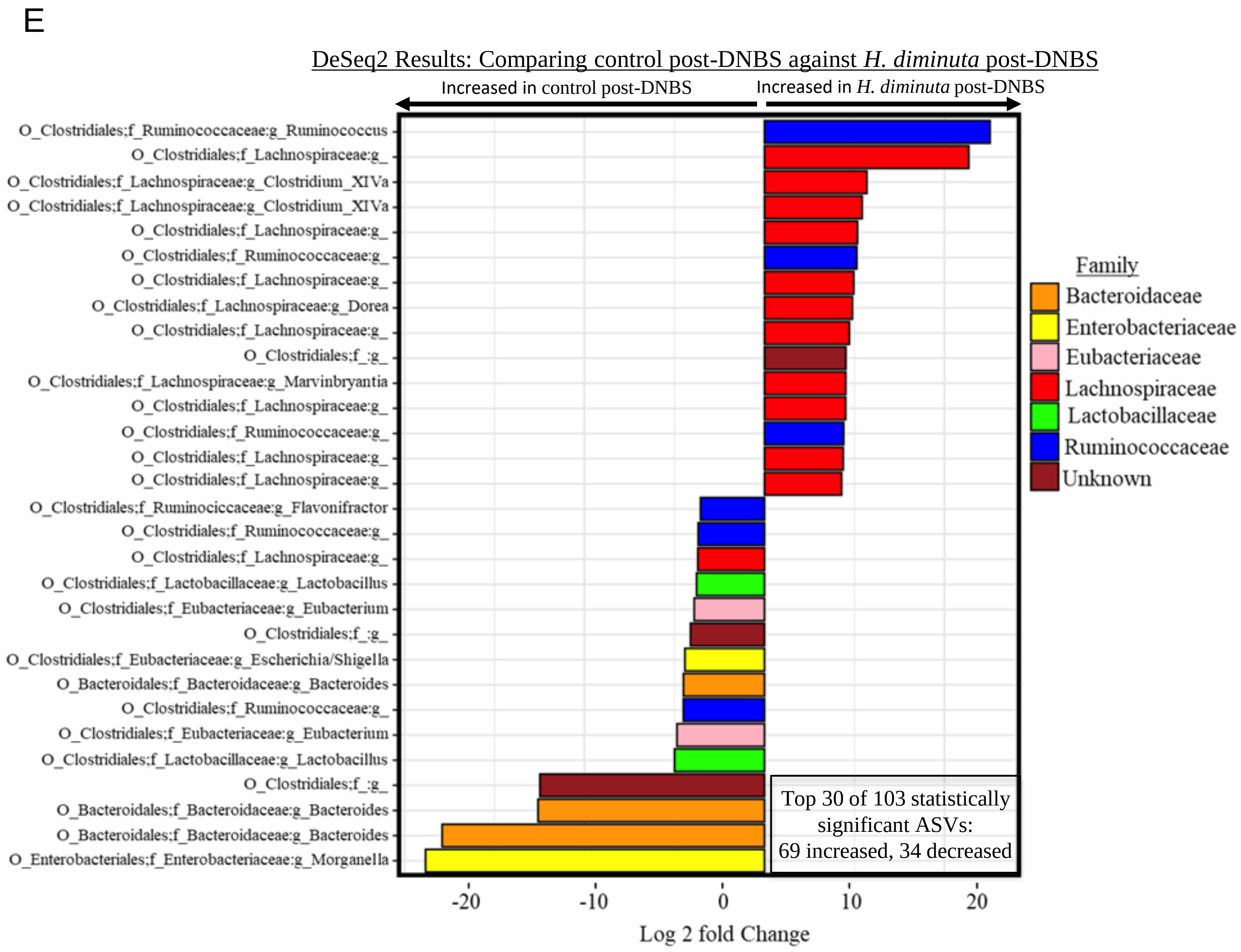

Shute et al 2021, Supplementary Figure 7

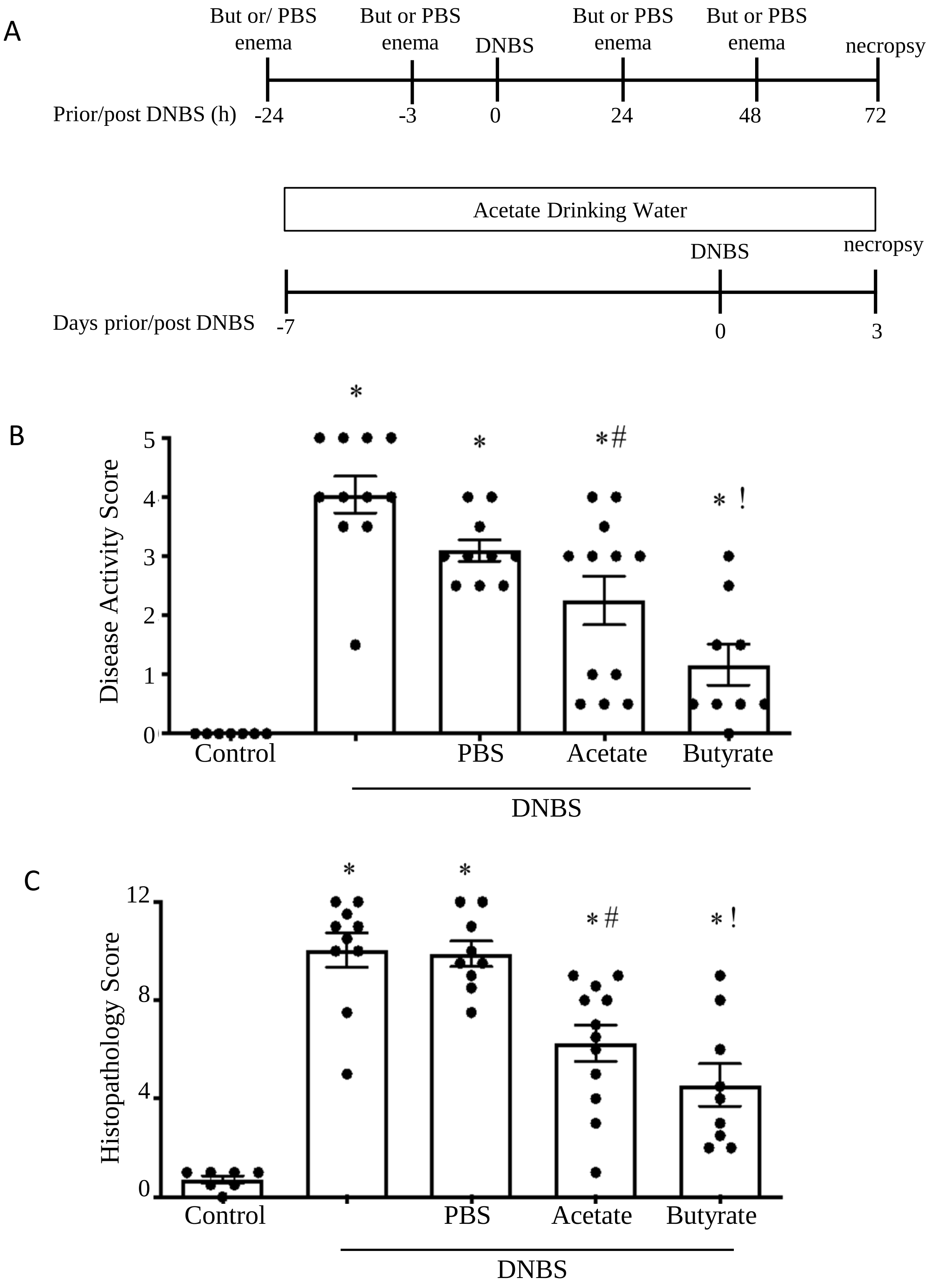

Shute et al 2021 Supplementary Figure 8

A

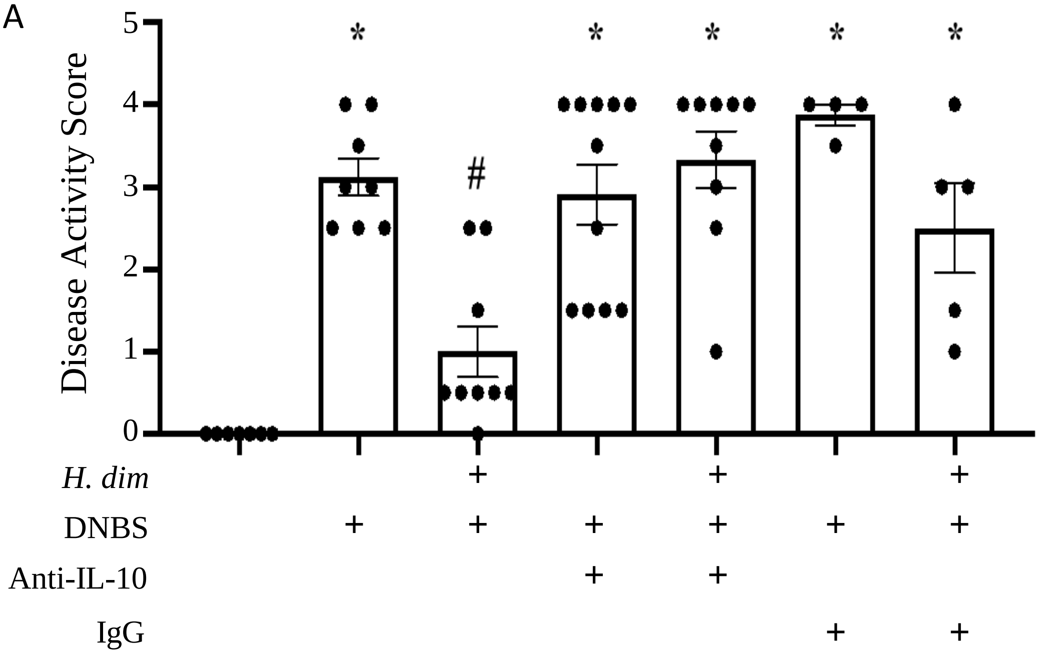

B

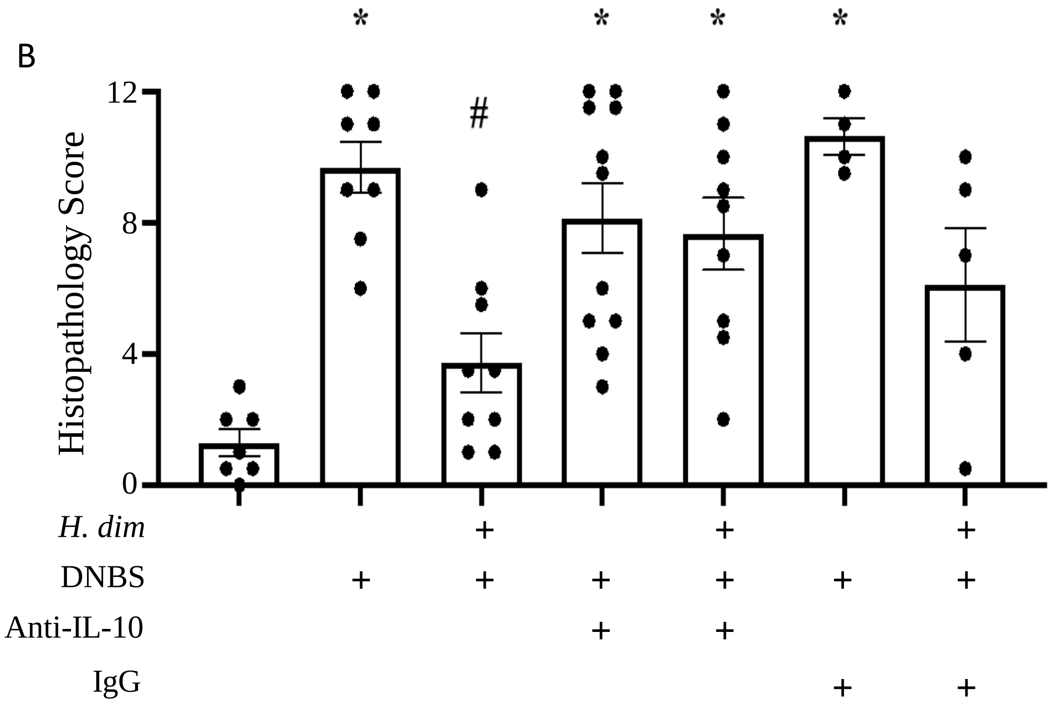

Supplement: Supplementary file 1 — Additional file 1: Suppl. Figure 1. Representative images of H&E stained sections of mid-colon from mice treated with broad-spectrum and DNBS. Suppl. Figure 2. Vancomycin or a combination of polymyxin B + neomycin impact gut bacteria in H. diminuta ± DNBS-treated mice. Suppl. Figure 3. Treatment with selected antibiotics interferes with H. diminuta-evoked inhibition of colitis. Suppl. Figure 4. Treatment with selected antibiotics affects stimulated splenic il-10 output. Suppl. Figure 5. H. diminuta-infected germ-free (GF) mice are not protected from DNBS-induced colitis. Suppl. Figure 6. Germ-free (GF) mice colonized with feces from H. diminuta-infected retain a distinct bacterial composition. Suppl. Figure 7. Short-chain fatty acids (SCFA) suppress DNBS-induced colitis. Suppl. Figure 8. Neutralizing anti-il-10 antibody block H. diminuta-evoked inhibition of DNBS-induced colitis. [file 40168_2021_1146_MOESM1_ESM.pdf]
