# Supplementary material for: Cooperation between host immunity and the gut bacteria is essential for helminth-evoked suppression of colitis
Source: Microbiome. 2021 Sep 13;9:186. doi: 10.1186/s40168-021-01146-2 (PMC8438845; doi:10.1186/s40168-021-01146-2)
Supplement: Supplementary file 2 — Additional file 2: Suppl. Table 1. Primer sequences used throughout this study. [file 40168_2021_1146_MOESM2_ESM.docx]

Supplementary Table 1: **Primer sequences used in mRNA analysis, bacterial community profiling and mouse genotyping**

| Target | Gene | Forward | Reverse | NCBI | |  |  |
| --- | --- | --- | --- | --- | --- | --- | --- |
| Mouse | il-10 | CGGGAAGACAATAACTGCACCC | CGGTTAGCAGTATGTTGTCCAGC | NM_010548 | |  |  |
| Mouse | il-10R | TTGCATACGGGACAGAACTGC | TCCAGGGTGAACGTTGTGAG | NM_008348.3 | |  |  |
| Human | MCT1 | TCCTTTTATCCTGCCACACC | GCATGCTGTTTTCCTTCTGC | AY364258.1 | |  |  |
| Mouse | mct1 | GTGACCATTGTGGAATGCTG | CTCCGCTTTCTGTTCTTTGG | NM_009196.4 | |  |  |
| Human | ABCG2 | CACCTTATTGGCCTCAGGAA | CCTGCTTGGAAGGCTCTATG | AY017168.1 | |  |  |
| Mouse | abacg3 | TCGCAGAAGGAGATGTGTTG | TTGGATCTTTCCTTGCTGCT | BC053730.1 | |  |  |
| Human | MCT4 | GCACCCACAAGTTCTCCAGT | CAAAATCAGGGAGGAGGTGA | BC112269.1 | |  |  |
| Mouse | mct4 | ACGGCTGGTTTCATAACAGG | CCAATGGCACTGGAGAACTT | BC046525.1 | |  |  |
| Human | HCAR2 | CTTATCTGGGCCCAACCTCTC | CTTGCAACCAGTCTCCCACT | NM_177551.4 | |  |  |
| Mouse | hcar2 | GAGCAGTTTTGGTTGCGAGG | GGGTGCATCTGGGACTCAAAT | NM_030701.3 | |  |  |
| Mouse | ifn-γ | TGCCAAGTTTGAGGTCAACAACCCA | CCCACCCCGAATCAGCAGCG | NM_008337.4 | |  |  |
| Mouse | ffar2 | CACCCAGAAGTTGGTCTGGT | GGGCAGGAAAATTCAGTCAA | NM_146187.4 | |  |  |
| Mouse | 18s rRNA | ACGCGCGCTACACTGACTGG | CGATCCGAGGGCCTCACTAAACC | NR_003278.3 | |  |  |
| Human | 18s rRNA | ATGGCCGTTCTTAGTTGGTG | CGCTGAGCCAGTCAGTGTAG | NR_003286.2 | |  |  |
|  | Universal Bacterial 16s Primers | | | | |  |  |
| Bacterial | 16s rRNA V3-V4 | CCTACGGGAGGCAGCAG | GACTACGCGGGTATCTAATCC | (1) | |  |  |
|  | Genotyping | | | | |  |  |
|  |  | Upstream from the gene | Within the gene | (2) | |  |  |
| Mouse | ffar2 | GCGGAAGTTGGATGCTGCTTCCACG | GCACAGTTCCTTGATCCTCACGGCC |  |  |  |  |
|  |  | Within the targeting cassette | |  |  |  |  |
|  |  | GGGCCAGCTCATTCCTCCCACTCAT | |  |  |  |  |
|  |  |  | | |  | |  |

1. Klindworth, A. et al. Evaluation of general 16S ribosomal RNA gene PCR primers for classical and next-generation sequencing-based diversity studies. Nucleic Acids Res. 41. 1–11 (2012).
2. Bjursell, M. et al. Improved glucose control and reduced body fat mass in free fatty acid receptor 2-deficient mice fed a high-fat diet. Am J Physiol Endocrinol Metab. 300. 211-220 (2011).
